# Effectiveness of Implementing a Collaborative Chronic Care Model for Clinician Teams on Patient Outcomes and Health Status in Mental Health: A Randomized Clinical Trial

**DOI:** 10.1001/jamanetworkopen.2019.0230

**Published:** 2019-03-01

**Authors:** Mark S. Bauer, Christopher J. Miller, Bo Kim, Robert Lew, Kelly Stolzmann, Jennifer Sullivan, Rachel Riendeau, Jeffery Pitcock, Alicia Williamson, Samantha Connolly, A. Rani Elwy, Kendra Weaver

**Affiliations:** 1 Center for Healthcare Organization & Implementation Research, VA Boston Healthcare System, Jamaica Plain, Massachusetts; 2 Department of Psychiatry, Harvard Medical School, Boston, Massachusetts; 3 Department of Biostatistics, Boston University School of Medicine, Boston, Massachusetts; 4 Department of Health Policy, Law, & Management, Boston University School of Public Health, Boston, Massachusetts; 5 Department of Anthropology, University of Iowa, Iowa City; 6 Behavioral Health Quality Enhancement Research Initiative Program, Central Arkansas Veterans Healthcare System, Little Rock; 7 School of Information, University of Michigan, Ann Arbor; 8 Department of Psychiatry and Human Behavior, Warren Alpert Medical School, Brown University, Providence, Rhode Island; 9 US Department of Veterans Affairs (VA) Office of Mental Health and Suicide Prevention, Washington, DC

## Abstract

**Importance:**

Collaborative chronic care models (CCMs) have extensive randomized clinical trial evidence for effectiveness in serious mental illnesses, but little evidence exists regarding their feasibility or effect in typical practice conditions.

**Objective:**

To determine the effectiveness of implementation facilitation in establishing the CCM in mental health teams and the impact on health outcomes of team-treated individuals.

**Design, Setting, and Participants:**

This quasi-experimental, randomized stepped-wedge implementation trial was conducted from February 2016 through February 2018, in partnership with the US Department of Veterans Affairs (VA) Office of Mental Health and Suicide Prevention. Nine facilities were enrolled from all VA facilities in the United States to receive CCM implementation support. All veterans (n = 5596) treated by designated outpatient general mental health teams were included for hospitalization analyses, and a randomly selected sample (n = 1050) was identified for health status interviews. Individuals with dementia were excluded. Clinicians (n = 62) at the facilities were surveyed, and site process summaries were rated for concordance with the CCM process. The CCM implementation start time was randomly assigned across 3 waves. Data analysis of this evaluable population was performed from June to September 2018.

**Interventions:**

Internal-external facilitation, combining a study-funded external facilitator and a facility-funded internal facilitator working with a designated team for 1 year.

**Main Outcomes and Measures:**

Facilitation was hypothesized to be associated with improvements in both implementation and intervention outcomes (hybrid type II trial). Implementation outcomes included the clinician Team Development Measure (TDM) and proportion of CCM-concordant team care processes. The study was powered for the primary health outcome, mental component score (MCS). Hospitalization rate was derived from administrative data.

**Results:**

The veteran population (n = 5596) included 881 women (15.7%), and the mean (SD) age was 52.2 (14.5) years. The interviewed sample (n = 1050) was similar but was oversampled for women (n = 210 [20.0%]). Facilitation was associated with improvements in TDM subscales for role clarity (53.4%-68.6%; δ = 15.3; 95% CI, 4.4-26.2; *P* = .01) and team primacy (50.0%-68.6%; δ = 18.6; 95% CI, 8.3-28.9; *P* = .001). The percentage of CCM-concordant processes achieved varied, ranging from 44% to 89%. No improvement was seen in veteran self-ratings, including the primary outcome. In post hoc analyses, MCS improved in veterans with 3 or more treated mental health diagnoses compared with others (β = 5.03; 95% CI, 2.24-7.82; *P* < .001). Mental health hospitalizations demonstrated a robust decrease during facilitation (β = –0.12; 95% CI, –0.16 to –0.07; *P* < .001); this finding withstood 4 internal validity tests.

**Conclusions and Relevance:**

Implementation facilitation that engages clinicians under typical practice conditions can enhance evidence-based team processes; its effect on self-reported overall population health status was negligible, although health status improved for individuals with complex conditions and hospitalization rate declined.

**Trial Registration:**

ClinicalTrials.gov Identifier: NCT02543840

## Key Points

**Question:**

Collaborative chronic care models for mental health conditions are supported by extensive randomized clinical trial data, but what is the evidence that these models can be implemented and can have beneficial effects in general clinical settings?

**Findings:**

In this randomized clinical implementation trial of 5596 veterans, a collaborative chronic care model was shown to be effectively implemented with practical, scalable facilitation support for clinicians. Effects on self-reported health outcomes were limited, but mental health hospitalization rate improved.

**Meaning:**

These findings suggest that collaborative chronic care models can be exported to general clinical practice settings using implementation facilitation and, at least for individuals with complex mental health conditions, can improve health outcomes.

## Teaser

This randomized clinical trial reports the process and outcomes of establishing the collaborative chronic care model in 9 mental health facilities within the US Department of Veterans Affairs system.

## Introduction

Collaborative chronic care models (CCMs) improve outcome in chronic medical conditions treated in primary care settings,^1,2,3^ depression treated in primary care facilities,^4,5^ and serious mental health conditions treated in mental health clinics.^6,7^ Collaborative chronic care models include several or all of 6 elements: work role redesign to support anticipatory, continuous care; self-management support; clinician decision support; clinical information systems; linkage to community resources; and leadership support.^3,8,9^ These elements are flexibly implemented according to local needs, capabilities, and priorities.^7^ However, little is known regarding how CCMs for mental health conditions perform when implemented in clinical practice with little to no exogenous research support.^10^

Data on implementing CCMs for mental health conditions come almost exclusively from depression treatment in primary care. Such CCMs can be successfully implemented,^11,12,13,14^ although their effect on clinical outcomes is inconsistent.^15,16^ The extent of implementation support provided in implementation trials has varied widely, in some cases including customized web-based tools, governance structures, and financial incentives.^11,13,17^ The sole CCM implementation controlled trial in mental health clinics used the Replicating Effective Programs framework^18^ compared with technical assistance to implement the Life Goals CCM^19^ for bipolar disorder and found better CCM implementation outcomes^20^ but no effect on clinical outcomes.^21^

Given the dearth of data on implementing CCMs in mental health clinics, we partnered with the US Department of Veterans Affairs (VA) Office of Mental Health and Suicide Prevention (OMHSP) to conduct a randomized implementation trial to establish CCM-based teams in 9 mental health clinics.^22^ We used a quasi-experimental,^23^ randomized stepped-wedge^24^ design to conduct a hybrid type II^25^ trial (ie, a trial focusing coequally on implementation and health outcomes). Such VA work has increasing relevance as health care systems and accountable care organizations move toward integrated care models.^26,27^ We hypothesized that implementation support, limited to an external facilitator working with facility staff, would enhance the adoption of CCM elements and improve veteran health outcomes.

## Methods

### Overview

This combined program evaluation and research project was reviewed by the VA Central Institutional Review Board (IRB). Implementation-related measures were exempt from IRB review, whereas veteran-level outcome measurement procedures were approved as research. Interviewed participants provided verbal informed consent, and a waiver was granted for patients studied through the administrative database only. The study rationale and design are detailed elsewhere.^22^ This study followed the Consolidated Standards of Reporting Trials (CONSORT) reporting guideline^28^ and the EQUATOR guidelines for reporting implementation research^29^ and cluster randomized clinical trials. The trial protocol can be found in Supplement 1.

In 2013, OMHSP began an initiative to enhance care coordination in general mental health clinics by establishing interdisciplinary teams in each VA medical center throughout the United States. Although OMHSP disseminated centrally developed guidance, it gave facilities broad latitude to develop team processes locally. In 2015, OMHSP adopted the CCM^6,7^ as the team model and partnered with study investigators to develop CCM implementation support. The VA health care system comprises more than 140 distinct facilities, including more than 1000 clinical sites caring for more than 5 million veterans annually. The initiative took place in general mental health clinics, which care for a mixed-diagnosis population, including those with mood, anxiety, and psychotic disorders, typically supported by specialty substance and posttraumatic stress disorder clinics.

### Site Selection

On the basis of veteran-level power calculations, 9 facilities were recruited from all VA facilities via publicity from OMHSP through the regional VA network leaders to individual mental health facility leaders. Qualifying facilities identified a general mental health clinic team, which committed to 1-hour weekly process redesign meetings for 12 months, and a staff member with process improvement experience to serve as an internal facilitator for 12 months at 10% effort (facility funded).

**Figure 1.**
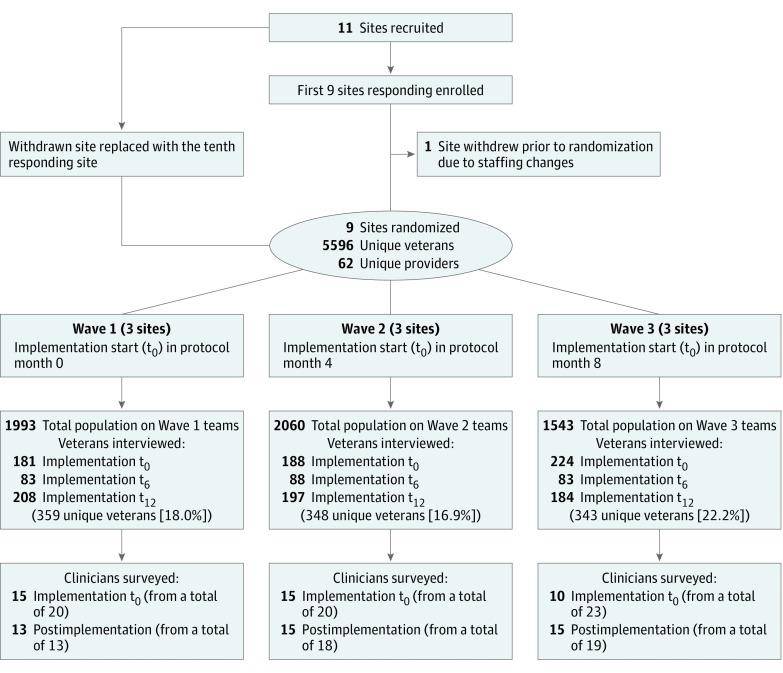
CONSORT Diagram for Facilities, Veteran Participants, and Clinicians Providers are mental health clinicians on the teams that received implementation support. The parenthetical t_0_ represents baseline prior to implementation of support; t_6_, the 6-month midpoint of implementation support; t_12_, postimplementation support after 12 months.

Twelve facilities qualified, and the first 9 to respond were enrolled. One site dropped out prior to randomization because of staffing changes and was replaced by the tenth facility (see Figure 1).

### Implementation Support

The implementation strategy was based on the model that health care is a complex adaptive system rather than a highly controlled machine.^22,30,31^ Specifically, implementation support was focused on creating conditions under which local solutions for local challenges could be developed in accordance with evidence-based guidance. This effort requires both team building, which has been shown to improve health care quality,^32,33,34,35,36,37,38,39,40^ and attention to specific health care processes. The CCM elements provided the clinical framework for process revision, considering the CCM as an integrated model, rather than a series of independent processes, based on the original insight by Wagner et al^9^ that improving single processes does not improve the outcome for chronic conditions. The blended internal-external facilitation model was chosen on the basis of evidence from previous VA work.^41^

Facilitation is a multifaceted strategy of interactive problem solving and support, which for this project included an external facilitator (study funded) who provided guidance and quality improvement expertise to teams while the internal facilitator (facility funded) helped on site to direct the implementation process.^42,43,44^ Facilitation was organized according to the 4 Replicating Effective Programs^45^ implementation stages: preconditions assessment, preimplementation preparation, implementation, and maintenance. Specific facilitation tasks included in-depth pre–site visit assessment and orientation of the site to the facilitation process and the CCM; 1½-day face-to-face site visit to launch the process redesign phase; 6 months of weekly videoconferences and/or conference calls with the team, weekly meetings with the internal facilitator, and ad hoc telephone and email communications; and 6 months of step-down facilitation contacts as needed.

Three of us (M.S.B., C.M., B.K.) each served as external facilitators for 3 sites, spending a mean (SD) 136.2 (27.6) hours per site in facilitation tasks for up to 12 months. Implementation activities were guided by a structured workbook that leads teams through self-assessment and process redesign according to the 6 CCM elements.

### Stepped-Wedge Design

**Figure 2.**
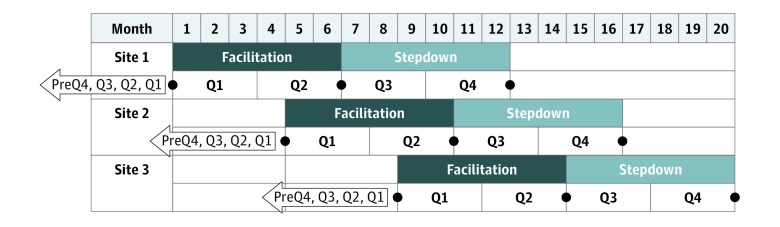
Protocol Structure: Implementation and Evaluation The implementation and evaluation protocol is illustrated for 3 facilities across 3 waves. Implementation consisted of 6 months of intensive facilitation followed by 6 months of step-down support (shaded rows). Facilities were assigned staggered start times for implementation, beginning at approximately 4-month intervals. The evaluative activities are illustrated beneath the implementation activities for each site (unshaded rows). Specifically, population-level hospitalization data were gathered on a quarterly basis from 12 months prior to the start of implementation (PreQ4-PreQ1) and for the 12 months of implementation (Q1-Q4). The veteran interview sample was assessed at the beginning of implementation, after 6 months, and after 12 months of implementation (black dots). Clinician assessment with the Team Development Measure took place at the beginning of facilitation and during step-down support. Thus, all evaluation activities were anchored to the start time of implementation support, considered protocol time zero (t_0_) for each site. Q indicates quarter of the year.

Given OMHSP’s desire for all participating facilities to receive implementation support, we used a stepped-wedge design.^24,46,47,48^ Specifically, facilities were randomized into 3 different start times of 3 sites each, with implementation support beginning at approximately 4-month intervals for each wave (start dates: February 2016 through February 2017; last site completed February 2018; Figure 2). To minimize imbalance in site characteristics across waves, we used a previously described algorithm^22^ to determine the least imbalanced randomization allocation schemas and then randomly selected 1 allocation scheme from among these. The control condition consisted of waiting for implementation support, during which time the facilities had continued access to OMHSP materials, received the CCM workbook, and participated in monthly technical support conference calls.

### Participant Population and Sample

The veteran population of interest consisted of individuals who were actively treated by a participating team, defined as at least 2 visits to a team clinic over the year prior to facilitation, including 1 visit in the previous 3 months. Only veterans with a diagnosis of dementia were excluded. This population served as the basis for hospitalization data analyses. From this population, 500 veterans from each facility were randomly selected. From these veterans, 85 were recruited for telephone interviews assessing health status and satisfaction at baseline, 6 months, and 12 months of implementation support. Clinical and demographic characteristics of the veterans included are summarized in Table 1, including race/ethnicity since they have been associated with mental health outcomes; race/ethnicity was characterized through administrative or interview data, both based on self-report. The clinician population included all members of the participating teams.

**Table 1.** Baseline Veteran Participant Characteristics

Variable	No. (%)		Population vs Sample Differences, χ^2^ or *F* Test	*P* Value	Effect Size^a^
	Population in Treatment With CCM Team (n = 5596)	Interviewed Sample (n = 1050)			
Demographic data					
Age, mean (SD), y	52.2 (14.5)	53.5 (14.0)	9.13	.003	0.0016
Female sex	881 (15.7)	210 (20.0)	15.9	.001	0.0522^b^
Race/ethnicity			3.6	.16	0.0256
White	4192 (79.5)	825 (81.7)			
Black	933 (17.7)	159 (15.7)			
Other	149 (2.8)	26 (2.6)			
Hispanic	676 (12.4)	97 (9.5)	9.1	.003	0.0400
Minority	1725 (31.3)	275 (26.5)	12.6	.001	0.0468
Married	2602 (46.8)	487 (46.7)	0.03	.86	0.0023
Employed (full- or part-time; self-employed)	2702 (50.2)	532 (52.5)	2.0	.16	0.0190
Rural residence	1205 (21.6)	227 (21.6)	0.03	.87	0.0022
Period of service, Gulf War or later (1990-present)	2858 (51.1)	497 (47.3)	6.7	.01	0.0339
VA disability ≥50%	4338 (77.5)	844 (80.4)	5.6	.02	0.0309
Clinical data					
Depression or anxiety disorder	3278 (58.6)	592 (56.4)	1.1	.30	0.0136
Serious mental illness (bipolar spectrum or schizophrenia)	1924 (34.4)	340 (32.4)	1.2	.27	0.0146
Posttraumatic stress disorder	2793 (49.9)	474 (45.1)	8.5	.01	0.0382
Substance use disorder	1096 (19.6)	169 (16.1)	8.1	.01	0.0373
No. of active mental health diagnoses, mean (SD)	2.3 (1.3)	2.2 (1.4)	4.8	.03	0.0008
No. of active medical diagnoses, mean (SD)	1.1 (1.3)	1.2 (1.3)	8.9	.03	0.0015
Mental health hospitalization in previous year	248 (4.4)	36 (3.4)	2.5	.11	0.0207
Medical-surgical hospitalization in previous year	405 (7.2)	73 (7.0)	0.1	.73	0.0046

Abbreviations: CCM, collaborative chronic care model; VA, US Department of Veterans Affairs.

^a^ Effect sizes for continuous variables are reported as η^2^, and categorical variables as Cramer V.

^b^ Purposive oversampling for representation of women.

### Implementation Outcome Measures

The implementation outcomes focused on team function plus concordance of team processes with the 6 CCM elements.^22,30,31^ Team function was assessed by clinician survey at baseline and during the second 6 months of implementation using the Team Development Measure,^49^ which consists of 4 subscales for rating items on a strongly agree to strongly disagree continuum: communication, cohesion, role clarity, and team primacy (prioritizing team over individual goals). Subscale scores were calculated as the mean percentage of subscale items endorsed as agree or strongly agree across clinicians.

Concordance of team processes with the CCM process was derived from the CCM workbook, which deconstructs the 6 CCM elements into 27 specific processes. Workbook process summaries were collected from each team at the end of the implementation support period (preimplementation process documentation was not required). We reviewed each process for each team and came to consensus ratings for each process of 0 (not consistent with the CCM or not completed), 0.5 (partially consistent), or 1.0 (fully consistent). Ratings for the 27 processes were compiled, and a percentage of CCM concordance across the 27 possible processes was calculated for each team.

### Intervention Outcome Measures

Self-rated veteran health status and satisfaction data were gathered by telephone interview of consented participants at baseline, 6 months, and 12 months of implementation support. The interviews included the mental component score (MCS) and physical component score of the Veterans RAND 12-item Health Survey,^50^ a measure adapted from the Short Form-12^51^ for health status (US general population mean [SD] score, 50 [10], with a higher score indicating better), and the short-form Quality of Life Enjoyment and Satisfaction Questionnaire^52^ for recovery-oriented quality of life (score range: 0-100, with a higher score indicating better). Care satisfaction was measured with the Satisfaction Index^53^ (score range: 12-72, with a higher score indicating better), and perception of collaborativeness of care was measured using the Patient Assessment of Chronic Illness Care^54^ (score range: 1-33, with a higher score indicating better). The primary intervention outcome variable, for which the study was powered, was the MCS.

Using the entire team-treated population, we gathered mental health hospitalization data from the VA Corporate Data Warehouse, as was done previously.^55^ Data were gathered on a quarterly basis from 1 year prior to implementation support (protocol t_-12_ to t_0_ [time zero]) through 1 year of implementation support (protocol t_0_ to t_12_), with a binary-coded variable for each veteran for each quarter (hospitalized or not).

### Statistical Analysis

Demographic and clinical characteristics of the overall population and interview sample were compared using unpaired, 2-tailed *t* test or χ^2^. For veteran self-report measures, the initial plan sought to randomly sample 85 veterans per team (90% power; α = .05; effect size, 0.20 for the MCS) for interview t_0_, t_6_, and t_12_, with as many veterans providing repeated measures as possible. When previously interviewed veterans were unavailable, additional randomly selected team-treated veterans who met the study criteria were interviewed in their stead, yielding a repeated cross-sectional design.^23^ Given the interviewer workflow, we reduced the t_6_ interview target sample size to 25; this reduction did not affect power for primary t_0_-to-t_12_ analyses.

For veteran interview data, a mixed-effects model was used, assuming that the scores were continuous with an underlying multivariate normal model. Linear contrasts of changes t_0_ to t_12_ composed primary analyses. To conduct subgroup analyses, we used interaction terms in the full model rather than stratification.^56^ The model used site as a random effect and the demographic and included clinical covariates listed in Table 1. The data on outcomes and major covariates were reviewed to detect excessive missing values, outliers, and severe skewness. Implementation start-time wave was collinear with time and hence excluded from the models.

Analyses were done with the SAS statistical package, version 9.4 (SAS Institute). Unadjusted and adjusted models yielded similar results, and both results are presented for key findings. All significance testing was 2-sided, with a statistical significance level that was Bonferroni corrected to *P* ≤ .01. Data analysis of this evaluable population was performed from June to September 2018.

## Results

### Study Flow and Participant Characteristics

The veteran population (n = 5596) included 881 women (15.7%), and the mean (SD) age was 52.2 (14.5) years. The interviewed sample (n = 1050) was similar but was oversampled for women (n = 210 [20.0%]). Sixty-two clinicians (35 women [56.5%]; mean [SD] age, 49.6 [9.9] years) participated.

Facility, veteran, and clinician participation are summarized in Figure 1. Comparison of veterans who consented to an interview and the population from which they were drawn are summarized in Table 1. Because of the large sample size, small but statistically significant differences were seen in several variables; however, effect sizes for the magnitude of these differences were negligible. Self-report data were missing for 9.8% of items across all measures. Intraclass correlation 1 values for the 5 clinical interview measures ranged from 0.0007 to 0.0296.

### Implementation Outcomes

Team Development Measure subscales (Table 2) showed high ratings for cohesion and communication at baseline, which did not change with implementation support. However, role clarity (53.4%-68.6%; δ = 15.3; 95% CI, 4.4-26.2; *P* = .01) and team primacy (50.0%-68.6%; δ = 18.6; 95% CI, 8.3-28.9; *P* = .001) improved statistically significantly. Teams varied on the proportion of the CCM-concordant processes achieved by the end of implementation support, ranging from 44% to 89% (eTable in Supplement 2). Postimplementation CCM process concordance and Team Development Measure scores, although measuring distinct implementation outcomes, were highly correlated (*r* = 0.90; *P* = .001).

**Table 2.** Summary of Implementation and Clinical Intervention Outcomes

Measure	Preimplementation, %	Postimplementation, %	Change, % (95% CI)	*P* Value
Implementation outcomes: Team Development Measure^a^				
Cohesion	84.0	84.5	0.5 (−7.4 to 8.4)	.75
Communication	83.3	84.4	1.1 (−5.6 to 7.7)	.90
Role clarity	53.4	68.6	15.3 (4.4 to 26.2)	.01
Team primacy	50.0	68.6	18.6 (8.3 to 28.9)	.001
Clinical intervention outcomes^b^				
VR-12				
MCS	30.7	30.9	0.2 (−1.3 to 1.5)	.97
PCS	42.5	43.7	1.2 (0.04 to 2.3)	.04^c^
QLESQ	49.8	50.3	0.5 (−1.3 to 2.3)	.58
Satisfaction index	53.0	52.4	−0.6 (−2.0 to 0.9)	.44
Patient assessment of chronic illness care	22.0	22.0	0.0 (−0.6 to 0.8)	.84

Abbreviations: MCS, mental component score; PCS, physical component score; QLESQ, Quality of Life Enjoyment and Satisfaction Questionnaire; VR-12, Veterans RAND 12-item Health Survey.

^a^ Subscale scores are the mean percentage of subscale items endorsed as agree or strongly agree across clinicians.

^b^ See Methods section in text for details.

^c^ Not significant after Bonferroni correction.

### Intervention Outcomes

#### Clinical Interview Measures

The MCS, the primary intervention outcome, did not change statistically significantly with implementation support in adjusted or unadjusted models, nor did other interview measures (Table 2). In post hoc analyses, those with complex clinical presentations, defined as receiving treatment for 3 or more mental health diagnoses in the previous year, demonstrated statistically significant improvements in MCS during the facilitation year (21.2-24.3; δ = 3.1; 95% CI, 1.0-5.3; *P* = .004), whereas those with 2 or fewer diagnoses declined nonsignificantly during that time (33.9-32.0; δ = –1.9; 95% CI, –3.7 to –0.1; *P* = .04). A linear contrast comparing the difference in MCS from t_0_ to t_12_ between those with a complex clinical presentation and those without was statistically significant (β = 5.03 [95% CI, 2.24-7.82; *P* < .001]; unadjusted β = 4.60 [95% CI, 1.90-7.29; *P* < .001]).

#### Mental Health Hospitalization Rate

**Figure 3.**
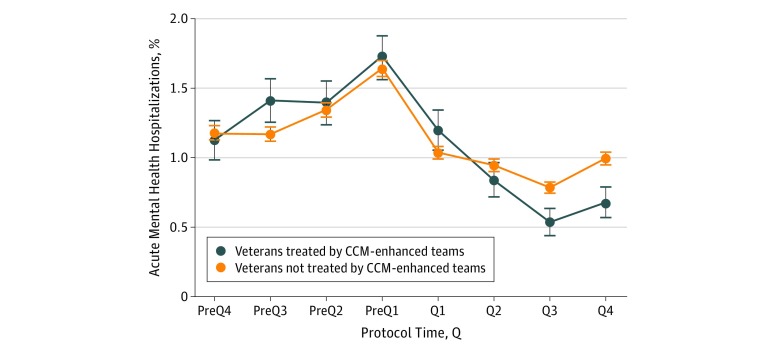
Mental Health Hospitalization Rates The x-axis displays protocol time, with implementation support occurring from Q1 through Q4. The blue line represents hospitalization rate for veterans treated by collaborative chronic care model (CCM)–enhanced teams, and the orange line represents veterans from the same clinics who were not treated by the CCM-enhanced teams (see text for details). Error bars represent SEs. Q indicates quarter of the year.

Implementation support was associated with statistically significant and sustained reduction in mental health hospitalization rate (β = –0.12 [95% CI, –0.16 to –0.07; *P* < .001]; unadjusted β = –0.11 [95% CI, –0.14 to –0.07; *P* < .001]; Figure 3). In post hoc analyses, we saw no difference in the way veterans were treated between higher- and lower-implementing teams (eTable in Supplement 2) in either hospitalization rate or MCS.

Given that this quasi-experimental trial did not include a separate control group, we addressed 4 threats to the internal validity of this finding. First, seasonal effects were deemed unlikely as waves began implementation support (protocol t_0_ in Figure 1) across the year.

Second, we considered that veterans who were hospitalized in the prefacilitation year might be less likely to stay enrolled in VA care during facilitation, thus decreasing population hospitalization risk at follow-up. More than 95% of veterans who receive mental health services do not receive mental health services outside of the VA system.^57^ We nonetheless reran the analyses, excluding those veterans who did not receive VA outpatient mental health care during implementation support. Only 435 veterans (7.8% [435 of 5596]) were excluded, and the findings remained statistically significant (β = –0.11; 95% CI, –0.15 to –0.06; *P* < .001).

Third, we investigated the unmeasured facility effects, independent of implementation support, by running identical analyses on veterans who met the study criteria but were treated in any mental health clinic, exclusive of the designated team’s clinics (n = 46 755). A linear spline model with a knot at t_0_ was used, and an interaction term was added to indicate whether the team-treated population change from before to after baseline (t_0_) differed from the analogous change for the non–team-treated population. As did the team-treated veterans, the non–team-treated veterans also demonstrated a statistically significant decrease in hospitalization rate during the facilitation period (β = –0.05; 95% CI, –0.07 to –0.04; *P* < .001; Figure 3). When the model included the interaction effect of team-treated and non–team-treated status in the spline model, the difference between team-treated and non–team-treated decrease in hospitalization rates was statistically significant (β = 0.19; 95% CI, 0.06 to –0.29; *P* < .001), with a larger decrease in hospitalization rate among team-treated veterans. During the facilitation year, the team-treated rate declined from 1.04% per quarter to 0.53% per quarter with a mean rate of 0.81% per quarter compared with a mean rate of 0.94% per quarter for the non–team-treated rate, a difference of 13.8% per quarter.

Fourth, we wondered whether the decrease in hospitalization rate concurrent with the start of facilitation at t_0_ (PreQ1 to Q1 in Figure 3) could be associated with simply having had an outpatient visit in PreQ1 (which was part of the team-treated population definition). Therefore, we investigated whether a decrease in hospitalization rate in a given quarter was more generally associated with a visit in the previous quarter. Specifically, we determined whether veterans with a visit in PreQ2 (n = 2089) demonstrated a statistically significant decrease in hospitalization rate from PreQ2 to PreQ1. They did not (1.15% to 1.05%; δ = 0.96%; 95% CI, –0.44 to 0.63; *P* = .72).

## Discussion

To our knowledge, this trial is one of the first studies to evaluate CCM implementation for individuals treated in mental health clinics and the first CCM trial to assess implementation effect in a multidiagnosis mental health population. The stepped-wedge design preserved scientific rigor while serving the policy needs of the health care system. The trial used minimal study-funded support (<3 hours per week per site of external facilitation effort), enhancing external validity and potential for scale-up and spread. Although the effect on overall population health status was negligible, facilitation improved team function and reduced population-level hospitalizations. These results align in general with those in previous CCM implementation trials showing improvements in implementation outcomes^11,12,13,14,19^ with mixed effects on clinical outcomes.^15,16,20^

Although MCS, the primary outcome, did not improve in the overall sample, those with more complex clinical symptoms did improve by a magnitude of 0.31 SD, comparable to meta-analyses on CCM clinical trial samples.^6^ The finding of a preferential effect in more complex individuals is consistent with evidence from an earlier VA multisite effectiveness trial^58^ and a recent meta-analysis of implementing patient-centered medical homes, which are based on CCM principles,^59^ which both demonstrated more benefit among higher-morbidity individuals.^60^ Also consistent with these findings are those from the previously noted earlier effectiveness trial that demonstrated reduced weeks in full-episode but not mean symptom levels.^61,62^ The primary population-based approach taken in the current study included less severely ill veterans, which may have mitigated the measured effect of care reorganization. The population-level effect on hospitalization rates may be the result of the substantial focus of implementation facilitation on changing workflow to support coordinated, continuous care. Overall, the results indicate that clinical benefits may be limited to those with complex clinical presentations or at risk for hospitalization.

We did not see differences in MCS or hospitalization rate between higher- and lower-implementing facilities, possibly because a critical threshold in CCM implementation had been met by all facilities; because chosen measures did not play a role in health outcome effects; or because specific individual CCM elements were responsible for improved outcome, which was not reflected in the overall CCM concordance measure. Exploring such mechanisms is the focus of ongoing mixed-methods analyses.

Taken together, the results suggest that implementation efforts at the clinician level enhance evidence-based care organization, which may result in improvements in outcome for more complex individuals and those at risk for hospitalization but no change in health status for the overall clinic population. However, more intense clinician-level support or simply more time may be needed to see population-level health status effects.

### Limitations

The major limitation of this study is the lack of an independent control group owing to health care system policy priorities and trial practicalities.^22^ However, the study design incorporated strengths recommended for quasi-experimental designs, including partial randomization, balancing of sites, embedding data collection at critical protocol points, and use of baseline measures.^23^ In addition, the hospitalization rate analyses employed multiple baseline and follow-up time points, and the finding that hospitalization rate decreased with facilitation withstood 4 tests of internal validity. Furthermore, for pragmatic reasons, the protocol did not include interview assessments at all facilities at all stepped-wedge time points; however, sufficient data were gathered to conduct the primary analysis.

The trial engaged only volunteer facilities within the VA. However, facilities enrolled did not differ substantially from the national VA facility pool in any characteristics identified a priori to balance the start-time waves.^22^ Furthermore, external validity limitations of VA-based studies are diminishing as health care organizations move toward integrated care models.^3,26,27,45^ Other limitations include small but statistically significant differences between the interviewed sample and the clinic population, follow-up limited to 1 year, and measures that may not reflect the effects of care reorganization.

## Conclusions

Working solely at the clinician level with minimal study-funded support, we found that implementing the CCM can reduce hospitalization rates and, for complex individuals, improve health status. The next challenge is to target, scale up, and spread implementation for teams that treat populations who are most likely to benefit from CCM care.

## Notes

Supplement 1. **Trial Protocol**

Supplement 2. **eTable.** By-Facility Summary of Implementation Outcomes

Supplement 3. **Data Sharing Statement**
