# Supplementary material for: Effectiveness of Implementing a Collaborative Chronic Care Model for Clinician Teams on Patient Outcomes and Health Status in Mental Health: A Randomized Clinical Trial
Source: JAMA Netw Open. 2019 Mar 1;2(3):e190230. doi: 10.1001/jamanetworkopen.2019.0230 (PMC6484628; doi:10.1001/jamanetworkopen.2019.0230)
Supplement: Supplement 1. — Trial Protocol [file jamanetwopen-2-e190230-s001.pdf]

CENTRAL IRB PROTOCOL TEMPLATE

Hybrid Controlled Trial to Implement Collaborative Care in General Mental Health

(A project of the QUERI Program for Team-Based Behavioral Health)

QUERI #15-289

Funding Agency: QUERI

Principal Investigator/Study Chair: Mark S. Bauer, MD

Version 1 September 21, 2015

Version 2 Amended November 16, 2015

Version 3 Amended March 4, 2016

Version 4 Amended May 6, 2016

Version 5 Amended June 17, 2016

Version 6 Amended July 20, 2016

Version 7 Amended August 3, 2016

Version 8 Amended October 3, 2016

Version 9 Amended December 9, 2016

Version 10 Amended January 23, 2017

Version 11 Amended October 13, 2017

Version 12 Amended February 16, 2018

Version 13 Amended July 31, 2018

Abstract

Provide a summary of the study (recommended length: less than 500 words).

Based on an internal system-wide review of mental health services and the Mental Health Action Plan submitted to Congress in November, 2011, OMHO has undertaken an effort to establish Behavioral Health Interdisciplinary Program teams (**BHIPs**), which are intended to provide General Mental Health (**GMH**) care throughout VA. The BHIP goal is to build effective interdisciplinary teams, which will provide the majority of care for Veterans in GMH. It is now expected that every VAMC establish at least one BHIP in the current initial phase (begun in late FY2013), and that the effort scale-up subsequently. Not surprisingly, progress has been uneven.

In 2015 OMHO incorporated the collaborative chronic care model (**CCM**) as an evidence-based model by which to structure BHIPs. Consistent with BHIP goals, CCMs were developed to provide anticipatory, continuous, collaborative, evidence-based care. CCMs consist of 6 elements: delivery system redesign, use of clinical information systems, provider decision support, patient self-management support, linkage to community resources, and healthcare organization support. Replicating Effective Programs with External Facilitation (**REP-F**) has been shown to be effective in implementing complex care models, including CCMs for MH, both within and beyond VHA.

In conjunction with OMHO, we plan a project including both an operational program evaluation component (non-research) and a research component (see letter from Drs. Carroll and Weaver from OMHO). The overall **Specific Aim** of the project is to evaluate the impact of REP-F in implementing CCM-based BHIPs.

OMHO Operational Program (Non-Research) Evaluation Aim: At the invitation of leadership of volunteer VA medical centers, we will employ REP-F-based implementation to establish CCM-based BHIPs. We will then evaluate the effectiveness of the process by monitoring OMHO national BHIP clinical fidelity measures. The results of this program evaluation activity will be provided to OMHO and medical center leadership.

Research Aims & Hypotheses: We hypothesize that BHIP implementation using REP-F will be accompanied by higher provider ratings of the presence of CCM elements by front-line BHIP providers in qualitative analysis of semi-structured interviews. Secondarily, we will utilize standard qualitative analytic techniques of the provider interview data to identify barriers and facilitators to BHIP implementation. These provider-focused research activities are referred to as “Study 1” in the sections below. In addition, we hypothesize that BHIP implementation using REP-F will be accompanied by Veterans rating their care as more consistent with the CCM, and by improved Veteran health status. These Veteran-focused research activities are referred to as “Study 2” in the sections below.

Note that this CIRB application addresses only these two specific research activities, described as Study 1 and Study 2 under the “Research Aims & Hypotheses” immediately above. The facilitation evaluation activities, described under “OMHO Operational Program (Non-Research)

Evaluation Aim" above are operational activities undertaken at the request of the OMHO and are therefore NOT considered research (See letter from Drs. Weaver and Carroll Appendix 2). Since our original submission of this protocol, additional guidance from the Office of Research Oversight has become available regarding the interface of quality improvement program evaluation and research; this has led us to reclassify our Veteran assessments (Study 2) as research due to the extent of data collected. We have therefore submitted this revised protocol. Similar combined operational program evaluation plus research activities were undertaken as part of QUERI RRP #13-237/VA CIRB 13-48*, which is conducted under the regulatory oversight of the VA Central IRB.*

In terms of specific procedures, as part of the OMHO national BHIP rollout we will provide REP-F facilitation support to 9 volunteer VAMCs identified by OMHO. Due to limited resources, we will stagger the timing of REP-F facilitation support, starting three VAMCs at each of six-month intervals, with the start time randomly assigned in conjunction with OMHO. Each site will receive REP-F facilitation support for 12 months, and non-research program evaluation data will be collected at several points before, during and after that time.

Research interview data will be collected from consenting providers prior to and after 12 months of REP-F facilitation support (Study 1). Interview data will be analyzed qualitatively; utilizing both directed content analysis to identify CCM elements in use as well as grounded thematic analysis to identify implementation barriers and facilitators. Veteran interview data will be collected from consenting Veterans on three occasions: prior to REP-F facilitation support, midway through the 12 months of REP-F facilitation support, and at the conclusion of the 12 months of REP-F facilitation support (Study 2). These Veteran interview data will be analyzed quantitatively.

List of Abbreviations

BHIP: Behavioral Health Interdisciplinary Program, an OMHO-mandated system redesign for general mental health service delivery.

BAA: Business Associates Agreement.

CDW: Corporate Data Warehouse.

CAVHS: Central Arkansas Veterans Healthcare Systems-NLR located in North Little Rock, Arkansas.

CCM: Collaborative Chronic Care Model, an evidence-based model for system redesign developed for primary care and now evidence-based for mental health care across primary and specialty care systems; serves as the system redesign model for the investigators' work to establish BHIPs in volunteer VAMCs.

CDW: Corporate Data Warehouse

CHOIR: The HSRD Center of Innovation at VA Boston and the Bedford VAMC, The Center for Healthcare Organization and Implementation Research, formerly the HSRD Centers of Excellence at VABHS and Bedford, COLMR and CHQOER.

DUA: Data Use Agreement.

EMIC: Ethnographic Methods and Implementation Core, an internal VA transcription service located at the VA Iowa City Healthcare System.

GLM: General Linear Model analysis.

GMH: General Mental Health clinics, established at every VAMC and the typical portal of entry to mental health specialty services for most Veterans with mental health needs.

IIR: Investigator-Initiated Research, the HSRD equivalent of a “Merit” grant award.

OMHO: Office of Mental Health Operations (10-NC5). Note that this office was renamed to the Office of Mental Health and Suicide Prevention (OMHSP) in 2017. OMHO will be used throughout for historical accuracy.

PACIC: Patient Assessment of Chronic Illness Care.

PACT: Patient-Aligned Care Teams, the mandated system redesign effort to restructure service delivery in primary care.

PHI: Protected Health Information

PII: Personally identifiable information.

PVAMC: CPL. Michael J. Crescenz VA Medical Center (Philadelphia)

Q-LES-Q: Quality of Life, Enjoyment, and Satisfaction Questionnaire

QUERI: VA Quality Enhancement Research Initiative.

RCT: Randomized controlled trial.

REDCap: the Research Electronic Data Capture system

REP-F: Replicating Effective Programs with Facilitation, an evidence-based implementation strategy utilized to support system redesign efforts within and beyond VA.

RRP: QUERI Rapid Response Project funding mechanism.

SCRSSN: Scrambled social security number.

TBBH QUERI: QUERI Program for Team-Based Behavioral Health, a set of projects led by Co-Principal Investigators JoAnn Kirchner and Mark Bauer funded by QUERI to facilitate and investigate various aspects of team-based behavioral health care. This study is one of the projects.

VABHS: VA Boston Healthcare System.

VAWNYHCS: VA Western New York Healthcare System

VR-12: Veterans Rand 12-item Social Functioning measure.

Contents

Protocol Title: Hybrid Controlled Trial to Implement Collaborative Care in General Mental Health 8

1.0 Study Personnel 8

2.0 Introduction 12

3.0 Objectives 18

5.0 Study Procedures 23

5.1 Study Design 23

5.2 Recruitment Methods 30

5.3 Informed Consent Procedures 32

5.4 Inclusion/Exclusion Criteria 33

5.5 Study Evaluations 33

5.6 Data Analysis 34

5.7 Withdrawal of Subjects 36

6.0 Reporting 36

7.0 Privacy and Confidentiality 36

8.0 Communication Plan 37

9.0 References 38

Protocol Title: Hybrid Controlled Trial to Implement Collaborative Care in General Mental Health

1.0 Study Personnel

- Provide name, contact information, and affiliations/employee status for the following:

Principal Investigator/Study Chair:

Mark Bauer, MD
Affiliation: VA Boston Healthcare System
Employment Status: 8/8ths VA
Phone: 857-364-6380
Fax: 857-364-6140
Email: Mark.Bauer@va.gov
Mailing Address:
150 South Huntington Avenue (152M)
Boston, MA 02130

Co-Investigators:

Martin P. Charns, DBA
Role: Co-Investigator
Affiliation: VA Boston Healthcare System
Employment Status: 8/8ths VA
Phone: 857-364-4945
Fax: 857-364-6140
Email: Martin.Charns@va.gov
Mailing Address:
150 South Huntington Avenue (152M)
Boston, MA 02130

Anashua Rani Elwy, PhD
Role: Co-Investigator
Affiliation: VA Boston Healthcare System
Employment Status: 8/8ths VA
Phone: 857-364-4944
Fax: 857-364-4511
Email: Rani.Elwy@va.gov
Mailing Address:
150 South Huntington Avenue (152-C)
Boston, MA 02130

Robert Lew, PhD
Role: Co-Investigator
Affiliation: VA Boston Healthcare System
Employment Status: 8/8ths VA
Phone: 857-364-6278
Fax: 857-364-4424
Email: Robert.Lew2@va.gov
Mailing Address:
150 South Huntington Avenue (MAVERIC)
Boston, MA 02130

Christopher Miller, PhD
Role: Co-Investigator, Facilitation Intervention Lead
Affiliation: VA Boston Healthcare System
Employment Status: 8/8ths VA
Phone: 857-364-5688
Fax: 857-364-6140
Email: Christopher.Miller8@va.gov
Mailing Address:
150 South Huntington Avenue (152M)
Boston, MA 02130

Bo Kim, PhD
Role: Co-Investigator
Affiliation: VA Boston Healthcare System
Employment Status: 8/8ths VA
Phone: 857-364-4867
Fax: 857-364-6140
Email: Bo.Kim@va.gov
Mailing Address:
150 South Huntington Avenue (152M)
Boston, MA 02130

Jennifer L. Sullivan, PhD
Role: Co-Investigator
Affiliation: VA Boston Healthcare System
Employment Status: 8/8ths VA
Phone: 857-364-5298
Fax: 857-364-6140
Email: Jennifer.Sullivan@va.gov
Mailing Address:
150 South Huntington Avenue (152M)
Boston, MA 02130

Samantha L. Connolly, PhD
Role: Co-Investigator
Affiliation: VA Boston Healthcare System

Employment Status: 8/8ths VA
Phone: 857-364-5987
Fax: 857-364-6140
Email: Samantha.Connolly@va.gov
Mailing Address:
150 South Huntington Avenue (152M)
Boston, MA 02130

Michael Ruderman, DO, MPH
Role: Co-Investigator
Affiliation: VA Boston Healthcare System
Employment Status: 8/8ths VA
Phone: 508-583-4500
Fax: 857-364-6140
Email: Michael.Ruderman@va.gov
Mailing Address:
150 South Huntington Avenue (152M)
Boston, MA 02130

JoAnn Kirchner, MD
Role: Co-Investigator, LSI at Little Rock
Affiliation: Central Arkansas Veterans Healthcare Systems - NLR
Employment Status: 8/8ths VA
Phone: 501-267-1719
Fax: 501-257-1703
Email: JoAnn.Kirchner@va.gov
Mailing Address:
2200 Fort Roots Drive, Building 58
North Little Rock, AR 72114

Karen Drummond, PhD
Role: Co-Investigator
Affiliation: Central Arkansas Veterans Healthcare Systems - NLR
Employment Status: 8/8ths VA
Phone: 949-293-9223
Fax: 501-257-1703
Email: Karen.Drummond@va.gov
Mailing Address:
2200 Fort Roots Drive, Building 58
North Little Rock, AR 72114

Laura O. Wray, PhD
Role: Co-Investigator, LSI at VA Western New York HCS
Affiliation: VA Western New York Healthcare System
Employment Status: 8/8ths VA
Phone: 716-862-8598
Fax: 716-862-6734
Email: Laura.Wray@va.gov
Mailing Address:
3495 Bailey Avenue

Buffalo, NY 14215

David W. Oslin, MD
Role: Co-Investigator, LSI at Philadelphia VA Medical Center
Affiliation: CPL. Michael J. Crescenz VA Medical Center (Philadelphia)
Employment Status: 8/8ths VA
Phone: 215-823-5894
Fax: 215-823-4123
Email: Dave.Oslin@va.gov
Mailing Address:
3900 Woodland Avenue Mailstop 116
Philadelphia, PA 19104

Other Study Staff

Kelly Stolzmann, MS
Role: Programmer Analyst
Affiliation: VA Boston Healthcare System
Employment Status: 8/8ths VA
Phone: 857-364-5355
Fax: 857-364-6140
Email: Kelly.Stolzmann@va.gov
Mailing Address:
150 South Huntington Avenue (152M)
Boston, MA 02130

Jeffery Pitcock, MPH
Role: Data Analyst, Interviewer
Affiliation: Central Arkansas Veterans Healthcare System – NLR
Employment Status: 8/8ths VA
Phone: (501) 257-1969
Fax: (501) 257-1707
Email: Jeffery.Pitcock@va.gov
Mailing Address:
2200 Fort Roots Drive, Building 58
North Little Rock, AR 72114

Rachel Riendeau, BA
Role: Project Manager, Quantitative Data Lead
Affiliation: VA Boston Healthcare System
Employment Status: 8/8ths VA
Phone: 401-633-4369
Fax: 857-364-6140
Email: Rachel.Riendeau@va.gov
Mailing Address:
150 South Huntington Avenue (152M)
Boston, MA 02130

2.0 Introduction

A. Scientific Background

A.1. Gaps in Outpatient Mental Health Outcomes and Quality. Mental health conditions affect 46.6% of Americans during their lives, and impact 26.6% in any given year[1]. Outcome for mental health conditions is suboptimal, and care coordination is problematic both within and beyond VHA[2,3]. While it is difficult to link individual care processes to suboptimal outcomes across diagnoses, multicomponent care models that emphasize care coordination and evidence-based care have been shown to improve health outcomes for individuals across a variety of mental health conditions (Section A.3.).

Given the need to improve outcome and care coordination for mental health conditions, VHA has established norms for recovery-oriented mental health care throughout VHA4, and invested heavily in mental health over the past decade.[5] At Senate hearings in 2011 the Committee on Veterans' Affairs has asked for an assessment of the impact of this investment, as well as plans for maximization of these resources[5]. In parallel, the Office of Mental Health Operations (**OMHO**) commissioned a VHA-wide assessment of mental health care delivery, supplemented by a report by the VA Office of Inspector General in Spring, 2012, on mental health access[6]. These assessments found clear opportunities for improvement, particularly around access to and coordination of services (Drs. Mary Schohn & Kendra Weaver (OMHO), oral & written communications, February-March, 2013). Recognizing opportunities for improvement, OMHO launched an effort to enhance delivery of mental health care, focusing in particular on outpatient General Mental Health (**GMH**) care. GMH is the typical portal of entry for Veterans requiring specialty mental health services; for instance, in VISN 1 the number of Veterans seen in each VAMC's GMH clinics ranges from 3,500-8,000 per year (Dr. Craig Coldwell, VISN 1 MH Lead, oral communication, April, 2013).

A.2. Behavioral Health Interdisciplinary Program Teams (BHIPs). In 2012 OMHO began a high priority effort to enhance care coordination in GMH care with the ambitious goal of establishing at least one interdisciplinary GMH team in every VAMC, beginning in late FY2013. The intent is that this team-based structure will spread and become the norm for GMH care in VHA, as GMH care is not typically team-based. These teams, called Behavioral Health Interdisciplinary Program teams (**BHIPs**), do not utilize a single care model. Rather, BHIPs are operationalized at the facility level to achieve the goal of building effective, interdisciplinary GMH teams at all VHA facilities that will provide the majority of mental health care necessary for a panel of assigned Veterans (OMHO, loc. cit.). National BHIP foci include the provision of continuous access to recovery-oriented, evidence-based care, emphasizing population-based care, consistent with the VA's Handbook on Uniform Mental Health Services in Medical Centers and Clinics[4]. It is planned that each BHIP will care for a panel of ~1,000 Veterans with 5-7

FTEE, including a mixture of licensed independent practitioners, clerical support, and non-licensed clinical support personnel (OMHO, loc. cit.).

In 2012, several pilot BHIPs across 4 VISNs were commissioned with support from OMHO. In 2013-14, a collaborative learning program linked these pilot BHIPs with one VAMC from each VISN. Nationally, however, despite centralized dissemination support from OMHO (monthly technical assistance calls, extensive SharePoint materials, ad hoc consultation), not all VAMCs are making progress. Similar to the PACT roll-out, many sites are struggling with how best to form their teams and establish team-based care (Kendra Weaver, OMHO, written & oral communications, 2014).

Throughout these efforts, no single care model has been adopted, nor has a single implementation strategy. The advantage to this is that individual VAMCs have flexibility to respond to local conditions in pursuing OMHO goals. However, the challenge is that while the overall goals are clear, there is no certainty that VAMCs will employ evidence-based models—for either the care model intervention or the implementation strategy. Following the PARIHS implementation model, which identifies evidence, context, and facilitation as key considerations in the implementation process[7,8], we along with OMHO leaders (see letter Appendix 2) propose that OMHO efforts to establish BHIPs can be enhanced by an evidence-based care model that can be adapted to site-specific contextual factors when implemented with external facilitation to support local efforts.

A.3. Collaborative Chronic Care Models (CCMs) as an Evidence-Based Model for BHIPs. Collaborative chronic care models (**CCMs**) provide an evidence-based and well-operationalized—yet flexible—model that can address OMHO needs by informing the BHIP effort. CCMs, as initially articulated by Wagner and colleagues[9,10] and subsequently as part of the Robert Wood Johnson Improving Chronic Illness Care initiative[11], represent a model of care that consists of several or all of six components: work role redesign, use of clinical information systems, provider decision support, patient self-management support, linkage to community resources, and health care leadership and organization support [e.g.,9,10,12-14]. Examples of how CCM elements can be operationalized are provided in the following Table.

Multiple randomized controlled trials indicate that CCMs improve outcomes for various chronic medical illnesses[e.g.,12-14] and depression treated in primary care[15,16]. CCM principles have informed Primary Care-Mental Health Implementation efforts such as TIDES[e.g.,17]. Dr. Bauer's work in bipolar disorder was the first effort to establish CCMs in GMH care (rather than primary care), in both a VA Cooperative Study[18] and an NIMH-funded multi-site randomized controlled trial (**RCT**) in a staff-model HMO[19]. Both

CCM Goal: Anticipatory, Continuous, Evidence-Based, Collaborative Care via…				
**Work Role Redesign**	**Self-Management Support for Individuals in Treatment**	**Decision Support**	**Information Management**	**Community Linkages**
• Care Management<br>• Access-Driven Scheduling<br>• Activated Follow-Up	• Incorporation of the Individual's Values and Skills<br>• Shared Decision-Making<br>• Self-Management Skills<br>• Behavioral Change Interventions	• Provider Education<br>• Practice Guidelines<br>• Specialty Consultation	• ***Population***: Registry<br>• ***Provider:*** Reminders<br>• Outcome Tracking<br>• Feedback<br>• Integrated Care Plans	• Additional Resources<br>• Peer-Based Support
**Organizational Leadership and Support**				

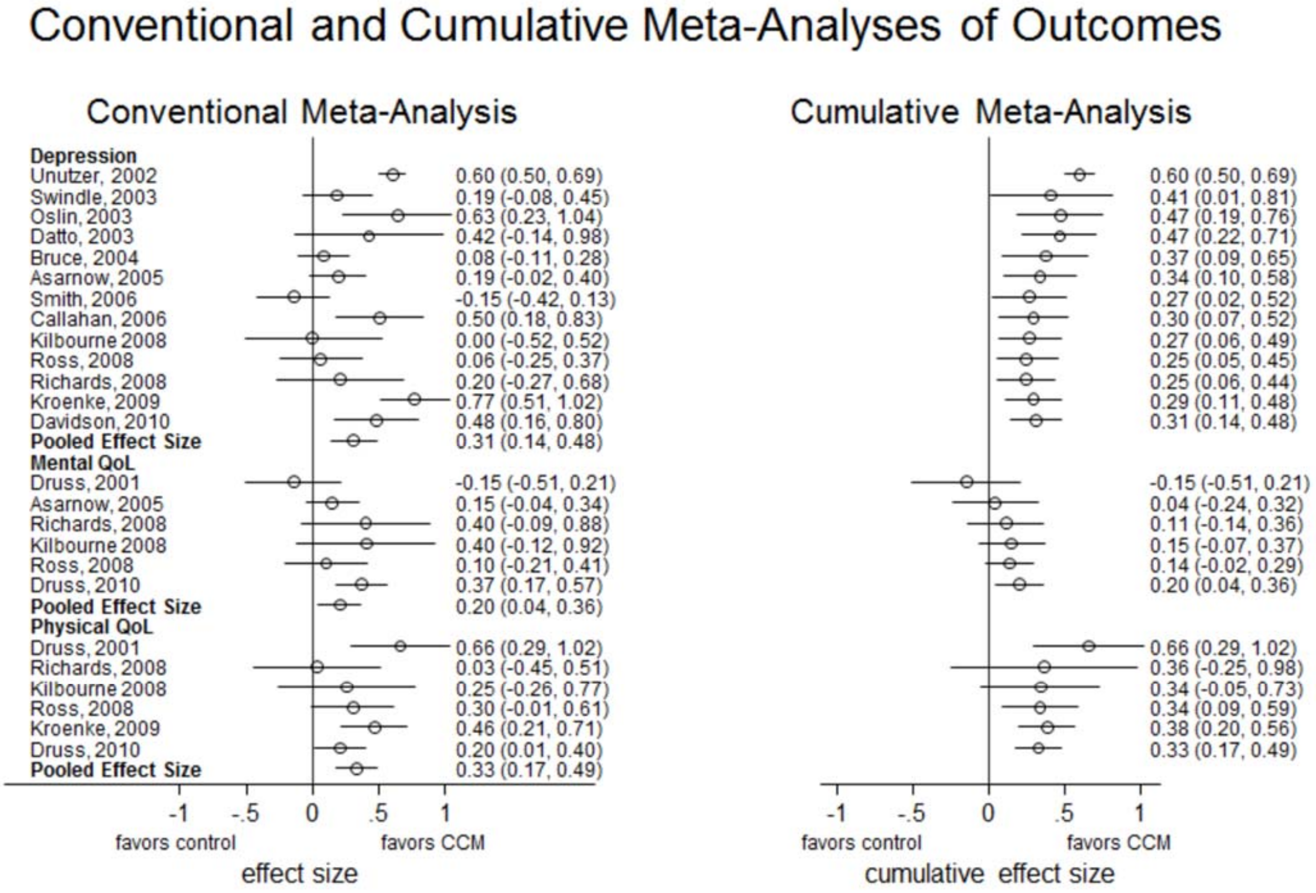

RCTs demonstrated that CCMs improved mental health outcomes at little[19] to no[18] added cost. Additional work has extended these findings, focusing on improving physical health outcomes in bipolar disorder[20-22], and broadening the model to mood disorders in general[23]. These efforts have resulted in the endorsement of CCMs in two national practice guidelines for bipolar disorder, including VA/DoD guidelines[24,25], and listing on the SAMHSA National Registry of Evidence-Based Programs and Practices[26]. Additionally, in 2011 the VA Office of Telehealth Services, supported by Mental Health Services and OMHO, established a telehealth version of the bipolar CCM, the Bipolar Disorders Telehealth Program, now active in 16 sites across 7 VISNs and 10 states with over 460 consults received. Thus the investigator team has extensive experience in establishing, adapting, and testing CCMs in both research and clinical contexts.

Of substantial relevance to BHIP efforts, our meta-analytic work indicates that CCMs have robust effects in a variety of mental health conditions and across both primary care and specialty care settings in RCTs[27]. Meta-analyses indicated significant CCM effects across disorders and care settings in depression, mental and physical quality of life, and social role function (d=0.20-0.33); total healthcare costs did not differ from controls. Note that the health outcome effect sizes likely indicate the lower bound for CCM effects, since the meta-analyses included only unadjusted analyses and included under-powered secondary analyses. Subsequent work with cumulative meta-analysis and meta-regression[28], indicates that the modal CCM in RCTs has employed the first four elements listed above, with no single element either essential or superfluous to the model. Moreover, effect sizes for depression and mental and physical quality of life have stabilized in the range of, respectively, 0.20 (0.04-0.36), 0.33 (0.17-0.49) and 0.31 (95%CI: 0.14-048)(Figure above)[28]. It should be noted that while a number of individual RCTs have enrolled mixed diagnostic groups[29-36], the majority have dealt with single conditions. Moreover, RCTs included in the above meta-analyses and published since show mixed results for conditions that typically require specialty-level care in VHA, particularly PTSD[36-39] and substance use disorders[40]. However, it is notable that these RCTs aimed to manage these conditions within primary or general care, while CCMs implemented in GMH could be oriented toward facilitating the transition of such patients to specialty programs while enhancing coordination of care.

A.4. Implementing the CCM: Replicating Effective Programs with External Facilitation (REP-F). In addition, we have done substantive work in the implementation of CCMs using the CDC's Replicating Effective Programs (**REP**) implementation strategy. REP was developed to translate effective HIV prevention strategies into practice for community-based organizations[41]. To our knowledge, we have been the first to apply REP strategies in healthcare organizations[42-44] both in establishing VA Bipolar Telehealth as a multi-VISN CCM, and as part of a two-state, five-site CCM implementation RCT (NIMH R-01-MH079994; PI: Amy M. Kilbourne).

We have chosen REP for our work from among many implementation frameworks because of its detailed articulation of concrete implementation actions arrayed across four stages: assessing Preconditions, Pre-Implementation preparation, Implementation, and post-implementation Maintenance. Major activities include development of site-specific Packaging of an intervention supported by provider Training and ongoing Technical Assistance to support intervention use. Classically, REP has focused primarily on maximizing fidelity to the

intervention[41], while our work has augmented REP with an **External Facilitation** component[45,46] to enhance provider buy-in and capability in order to establish and reinforce desired norms, and to achieve broader organizational support by identifying and aligning organizational incentives to the implementation efforts[42,43,45-48]. Specifically, external facilitators help providers to address organizational barriers and foster ongoing relationships with organizational leadership to promote buy-in by ensuring alignment of REP efforts to organizational goals and priorities[43,46-48].

In terms of preliminary data regarding REP in combination with External Facilitation, we randomized five sites in Michigan or Colorado to implement the bipolar CCM via REP with External Facilitation (**REP-F**) vs. REP with minimal technical assistance to support CCM use. After six months we assessed two types of fidelity monitors, focusing on specific, protocol-determined[18] components: treatment receipt and treatment competency. Sites receiving REP-F demonstrated 2.6-fold more total care management contacts and their patients were 7.2 times more likely to receive sessions with adequate treatment fidelity[44]. Thus REP-F demonstrates the ability to enhance fidelity to CCM in community practices, even compared to a “lighter” version of REP. Of additional importance, REP-F has also been shown to improve outcomes in a program geared to improving population-level outcomes in VA[49].

B. Significance

We are working in partnership with OMHO and QUERI to improve Veteran health status and quality of care by supporting the implementation of team-based collaborative care for Veterans with mental health conditions, and to support OMHO’s efforts to develop the most effective implementation strategy for doing so.

In summary, *our team is well positioned to assist OMHO in their efforts to implement CCM-based BHIP teams nationally. In discussions with OMHO, it was decided that the next logical step is to conduct a program evaluation with volunteer VAMCs that desire implementation assistance to establish their BHIP teams. Because of limited implementation workforce, and in view of OMHO’s desire for empirical evidence that this type of implementation support is worth their investment, it is planned to randomly assign sites to the timing of REP-F implementation support using a stepped wedge design [50,58-60]. Thus we will conduct a randomized program evaluation under the sponsorship of OMHO, supplemented by funding from VA Quality Enhancement Research Initiative (QUERI), which will provide implementation support utilizing REP-F and assess impact on national BHIP fidelity measures. We will also conduct a research study to assess BHIP provider perceptions of BHIP care according to the CCM elements (Study 1) and assess program impact on Veteran perceptions of collaborativeness of care and health status (Study 2).*

This project is clearly aligned with the highest priorities of OMHO, as articulated by Dr. Schohn in Senate hearings: “VHA’s actions prescribe mental health teams; staffing plans based on approved patient panel sizes; and measureable improvement of patient-centered outcomes for depression, PTSD, suicide, substance use, and mental health recovery.”[5] <u>Notably, this program evaluation effort represents a unique and time-sensitive opportunity to incorporate a highly</u>

evidence-based care model into the early implementation stages of a national initiative before the roll-out is completed. Since this project utilizes no externally funded staff beyond implementation support, results will be readily translatable into operational guidance for OMHO. Meanwhile, our work will provide a more intensive intervention for struggling sites. These synergistic efforts will place OMHO on even stronger footing to respond to diverse site needs as their nationwide initiative continues.

Regarding research contribution to implementation science, we recall that the fundamental insight that led to the development of CCMs was that improving individual care processes did not reliably improve health outcomes in target populations[9,10]. This is not surprising in light of evidence that healthcare represents a complex adaptive system rather than a simple mechanical system[51,52]. That is, healthcare represents a group of human beings working more or less together who "have the freedom and ability to respond to stimuli in many different and fundamentally unpredictable ways"[52,p310]. Thus the fundamental approach to change is not to specify behavior in more and more detail "to make the human parts act more mechanical"[52,p310] but rather to create the conditions under which clinician behavior develops solutions that will lead to more effective care. It will be extremely informative to characterize and analyze front-line participating providers' perceptions of the degree to which the CCM was successfully implemented, and the barriers and facilitators of its implementation.

C. Target Population including Vulnerable Populations

Study 1 (Providers)

Our research evaluation will sample providers based on BHIP membership. Regarding providers, we are well aware that they as employees represent a vulnerable population, having worked with this population in the IRB-approved Collaborative Care for Returning Veterans study [53], and will take care to prevent both coercion to participate and compromise of confidentiality.

Foundational work for the BHIP project was conducted during a QUERI-funded study, RRP #13-237, for which the VA Central IRB has regulatory oversight (VA CIRB 13-48). For that study, as well as the current project, the implementation processes are considered operations, and therefore not subject to IRB oversight, while the provider assessments are considered research (see letter from Drs. Weaver and Carroll of OMHO Appendix 2). Specifically, the formation of a BHIP team is an operational decision by an individual VAMC, and employees cannot necessarily opt in or out as part of the research process.

Study 2 (Veterans)

We will assess patient-level process and health outcome measures at the population level by utilizing the recruitment and telephone interview procedures as described below. For this strategy we will use Corporate Data Warehouse (**CDW**) data to identify all Veterans seen in the prior year by BHIP providers. We will exclude individuals with an encounter for a diagnosis of

dementia in the prior year, and determine how well the racial, ethnic, and gender distribution of the interviewed sample resembles that of the population from which it was drawn.

3.0 Objectives

A. Specific Aims

Based on an internal system-wide review of mental health services and the Mental Health Action Plan submitted to Congress in November, 2011, OMHO has undertaken an effort to establish Behavioral Health Interdisciplinary Program teams (**BHIPs**), which are intended to provide General Mental Health (**GMH**) care throughout VA. The BHIP goal is to build effective interdisciplinary teams, which will provide the majority of care for Veterans in GMH. It is now expected that every VAMC establish at least one BHIP in the current initial phase (begun in late FY2013), and that the effort scale-up subsequently. Not surprisingly, progress has been uneven.

In 2015 OMHO incorporated the collaborative chronic care model (**CCM**) as an evidence-based model by which to structure BHIPs. Consistent with BHIP goals, CCMs were developed to provide anticipatory, continuous, collaborative, evidence-based care. CCMs consist of 6 elements: delivery system redesign, use of clinical information systems, provider decision support, patient self-management support, linkage to community resources, and healthcare organization support. Replicating Effective Programs with External Facilitation (**REP-F**) has been shown to be effective in implementing complex care models, including CCMs for MH, both within and beyond VHA.

In conjunction with OMHO, we plan a project including both an operational program evaluation component (non-research) and a research component (see letter from Drs. Carroll and Weaver of OMHO). The overall **Specific Aim** of the project is to evaluate the impact of REP-F in implementing CCM-based BHIPs.

OMHO Operational Program (Non-Research) Evaluation Aim: At the invitation of volunteer VA medical centers, we will employ REP-F-based implementation to establish CCM-based BHIPs. We will then evaluate the effectiveness of the process by monitoring OMHO national BHIP clinical fidelity measures. The results of this program evaluation activity will be provided to OMHO and medical center leadership.

Research Aims & Hypotheses: We hypothesize that BHIP implementation using REP-F will be accompanied by higher provider ratings of the presence of CCM elements by front-line BHIP providers in qualitative analysis of semi-structured interviews. Secondarily, we will utilize standard qualitative analytic techniques of the provider interview data to identify barriers and facilitators to BHIP implementation. These provider-focused research activities are referred to as “Study 1” in the sections below. In addition, we hypothesize that BHIP implementation using REP-F will be accompanied by Veterans rating their care as more consistent with the CCM, and by improved Veteran health status. These Veteran-focused research activities are referred to as “Study 2” in the sections below.

Note that this CIRB application addresses only these two specific research activities, described as Study 1 and Study 2 under the "Research Aims & Hypotheses" immediately above. The other activities, described under "OMHO Operational Program (Non-Research) Evaluation Aim" above are operational activities undertaken at the request of the OMHO and are therefore NOT considered research (See letter from Drs. Weaver and Carroll Appendix 2). Since our original submission of this protocol, additional guidance from the Office of Research Oversight has become available regarding the interface of quality improvement program evaluation and research; this has led us to recognize that our patient assessments (Study 2) are more appropriately classified as research due to the extent of data collected. We have therefore submitted this revised protocol. Similar combined operational program evaluation plus research activities were undertaken as part of QUERI RRP #13-237, which is conducted under the regulatory oversight of the VA Central IRB (VA CIRB 13-48).

In terms of specific procedures, as part of the OMHO national BHIP rollout we will provide REP-F facilitation support to 9 volunteer VAMCs identified by OMHO. Due to limited resources, we will stagger the timing of REP-F facilitation support, starting three VAMCs at each of six-month intervals, with the start time randomly assigned in conjunction with OMHO. Each site will receive REP-F facilitation support for 12 months, and non-research program evaluation data will be collected at several points before, during and after that time.

Research interview data will be collected from consenting providers prior to and after 12 months of REP-F facilitation support (Study 1). Interview data will be analyzed qualitatively; utilizing both directed content analysis to identify CCM elements in use as well as grounded thematic analysis to identify implementation barriers and facilitators. Veteran interview data will be collected from consenting Veteran patients on three occasions: prior to REP-F facilitation support, midway through the 12 months of REP-F facilitation support, and at the conclusion of the 12 months of REP-F facilitation support (Study 2). These Veteran interview data will be analyzed quantitatively.

4.0. Resources and Personnel

A. Research Sites

Research will be conducted by investigators at VABHS, CAVHS, VAWNYHCS and PVAMC. All data will be collected by investigators at these sites and stored only at these sites. The nine VAMCs that have volunteered to utilize REP-F implementation methods to establish a BHIP based on CCM principles will not be engaged in research. We consider these 9 sites to be not engaged in the conduct of research, as described in the U.S. Department of Health and Human Services, OHRP, *Guidelines on Engagement of Institutions in Human Subjects Research*, particularly Section III.B, and as was the case for the Central IRB-approved RRP #13-237/VA CIRB 13-48 (http://www.hhs.gov/ohrp/policy/engage08.html).

Sites whose roles are limited to providing subjects for this research project are considered not engaged in research (though of course VABHS, CAVHS, VAWNYHCS, and PVAMC are engaged in research by virtue of their investigators on the study). Specifically, for Study 1 the

nine VAMCs will provide information about the study to the population of eligible providers. For Study 2, the nine VAMCs will provide relevant clinic names from which the Veteran sample will be drawn by investigators at VABHS. They will release information to the investigators on potential subjects, including identifiable information; however, the provision of such information is consistent with VAMC operational (non-research) duties, such as they would do for External Peer Review, OMHO reviews, and other internal business or clinical operational tasks. Their staff will not conduct interviews, provide interventions, or consent subjects. Furthermore, their role in providing information will not merit professional recognition or publication authorship of study results. We will notify facility leadership before we engage in any recruitment activities or conduct the telephone interviews.

For the prior RRP, the Central IRB determined that the participating VAMCs that request facilitation support to establish a BHIP are *not* engaged in research. This is because, while the site is providing information on the overall population of interest (employees involved in BHIP, BHIP clinics from which subjects are drawn), the actual sample recruitment will be done by investigators at the Boston and North Little Rock VAMCs. This decision follows the principles described in the U.S. Department of Health and Human Services, Office of Human Research Protection, *Guidelines on Engagement of Institutions in Human Subjects Research*, October 16, 2008, particularly Section III.B. Study 1 research activities, i.e. interviews with providers, will be conducted by investigators at the Boston and North Little Rock VAMCs and will fall under the purview of the Central IRB and the R&D Committees at those facilities. Study 2 research activities, i.e. telephone interviews with Veterans, will be conducted by research assistants located at VABHS and CAVHS, and will also fall under the purview of the Central IRB and the R&D Committees at those facilities.

B. Individuals and their Specific Roles in the Study

Study 1: No protected health information will be collected during the provider interviews so study team members will not have access to protected health information (**PHI**) for Study 1. The interview will ask about how care is organized and delivered in the General Mental Health clinic and does not include personal health questions. However, the team members will have access to personally identifiable information (**PII**) for Study 1 such as GMH employee names, positions and contact information. (Please see Section 7.0 Privacy and Confidentiality for data security safeguards.)

Study 2: Study 2 will involve collection of PHI as well as PII for Veterans.

Specific roles and permissions are summarized in this bulleted list and in the table below.

- **Mark S. Bauer, MD (PI, VABHS)** is an experienced health services researcher, implementation scientist, and VA administrator who has contributed to the CCM literature and the codification of hybrid designs. He serves as an Associate Director of the Boston-Bedford HSRD Center of Innovation, The Center for Healthcare Organization and Implementation Research (**CHOIR**). He will oversee all aspects of the evaluation,

will serve as an External Facilitator and consult on analysis of research and operations program evaluation data with the assistance of Rachel Riendeau, BA **(Project Manager, Quantitative Data Lead, VABHS)** and **Kelly Stolzmann, MS (Programmer Analyst, VABHS).** *Dr. Bauer will have access to provider PII (Study 1) and will have access to Veteran PHI and PII (Study 2). He will not consent subjects for Study 1 or Study 2. Ms. Riendeau and Ms. Stolzmann will have access to provider PII (Study 1) and Veteran PII and PHI (Study 2). Ms. Stolzmann will not consent subjects but Ms. Riendeau will consent Veterans (Study 2).*

- **Martin Charns, DBA (Co-I, VABHS)** is a senior investigator and the Co-Director Emeritus of CHOIR and a Professor in the Boston University School of Public Health. He will apply his expertise in organizational science and change to interpreting provider interview data. *He will not have access to provider PII (Study 1) or Veteran PII or PHI (Study 2) and will not consent subjects.*
- **A. Rani Elwy, PhD (Co-I, VABHS)** is an accomplished qualitative researcher at CHOIR and will oversee data analysis for the qualitative interviews and consult on implementation processes. *She will have access to provider PII (Study 1) but not Veteran PII or PHI (Study 2) and will not consent subjects.*
- **Jennifer Sullivan, PhD (Co-I, VABHS)** will conduct, code and analyze the provider interviews**.** *She will have access to provider PII and will consent provider subjects (Study 1) but will not have access to Veteran PII or PHI nor consent Veteran subjects for Study 2.*

- **Robert Lew, PhD (Co-I, VA Boston Cooperative Studies Coordinating Center)** is a senior biostatistician with extensive experience in clinical trials design and execution, including nested analyses and multi-level adjustment in GLM. He will consult on qualitative data analyses from the perspective of having participated in site balance during randomization. *He will not have access to provider PII (Study 1) but will have access to Veteran PII and PHI (Study 2) and will not consent subjects.*
- **Christopher J. Miller, PhD, (Co-I, VABHS)** is a clinical psychologist who completed his HSR&D post-doctoral fellowship under Dr. Bauer. He is experienced in qualitative interviews from several projects and has been a leader in developing CCM self-management program and provider education materials. He will serve as an External Facilitator and as the Facilitation Intervention Lead and contribute to qualitative data analyses. *He will have access to provider PII (Study 1) and will not consent subjects. He will have access to Veteran PII and PHI but will not consent subjects (Study 2)*
- **Bo Kim, PhD (Co-I, Boston)** is a former OAA Postdoctoral Fellow and is an investigator at CHOIR. She will serve as an External Facilitator and contribute to qualitative data analyses. *She will have access to provider PII (Study 1) but not Veteran PII or PHI (Study 2) and will not consent subjects*.
- **Samantha L. Connolly, PhD (Co-I, Boston)** is a current Postdoctoral Fellow at CHOIR and a licensed clinical psychologist. She will assist with qualitative and quantitative analyses (Study 1 and Study 2) and with conducting provider interviews as well as the telephone interviews with Veterans. She will conduct telephone interviews of Veterans (Study 2) and will have access to provider PII and will consent provider subjects (Study

1). She will have access to Veteran PII and PHI (Study 2) and will consent subjects for Study 2.

- **Michael Ruderman, Do, MPH (Co-I, Boston)** is a licensed psychiatrist and public health professional completing training in implementation science under mentors Dr. Kim and Bauer. He will assist with qualitative (Study 1) and quantitative (Study 2) and will have access to Provider PII and Veteran PII and PHI (Study 2).
- **JoAnn E. Kirchner, MD (Co-I, CAVHS)** is the Director of Mental Health QUERI and an expert in External Facilitation. She will provide ongoing consultation on External Facilitation during the study. *She will have access to provider PII (Study 1) and Veteran PII and PHI (Study 2) but will not consent subjects.*
- **Karen Drummond, PhD (Co-I, CAVHS)** will assist in conducting the provider interviews as well as the telephone interviews of Veterans. *She will have access to provider PII and will consent provider subjects (Study 1) and conduct telephone interviews of Veterans (Study 2). She will have access to Veteran PII and PHI (Study 2) and will consent subjects for Study 2.*
- **Jeffery Pitcock, MPH (Data Analyst, CAVHS)** is responsible for programming the REDCap database and data collection instruments for Study 2. He will have access to provider PII (Study 1) and Veteran PII and PHI (Study 2). He will also conduct telephone interviews of Veterans (Study 2) and will consent subjects.
- **Laura O. Wray, PhD (Co-I, VAWNYHCS)** is the director of the VA Center for Integrated Healthcare. She will serve as the Local Site Investigator and will supervise the VAWNYHCS research assistants in data collection. *She will have access to Veteran PII and PHI (Study 2) but will not consent subjects.*
- **David W. Oslin, MD (Co-I, PVAMC)** is the Director of the VISN 4 Mental Illness, Research, Education, and Clinical Center (MIRECC) and the Chief of Behavioral Health at the Philadelphia VAMC. He will serve as the Local Site Investigator and will supervise the PVAMC research assistant in data collection. *He will have access to Veteran PII and PHI (Study 2) but will not consent subjects.*

Study Staff Member	Study 1: Access to Provider PII?	Study 2: Access to Veteran PII or PHI?	Obtain consent from providers (Study 1) or Veterans (Study 2)?
Mark S. Bauer	Yes	Yes	No
Martin Charns	No	No	No
A. Rani Elwy	Yes	No	No
Rachel Riendeau	Yes	Yes	Study 2
Robert Lew	No	Yes	No
Kelly Stolzmann	Yes	Yes	No
Christopher Miller	Yes	Yes	No
Bo Kim	Yes	No	No
Jennifer Sullivan	Yes	No	Study 1
Samantha L. Connolly	Yes	Yes	Study 1, 2
Michael Ruderman	No	Yes	No
JoAnn Kirchner	Yes	Yes	No
Jeffery Pitcock	Yes	Yes	Study 2
Karen Drummond	Yes	Yes	Study 1, 2
Laura O. Wray	No	Yes	No
David W. Oslin	No	Yes	No

C. Contracting and Business Associates Agreement

The investigators have contracted with the Ethnographic Methods and Implementation Core (EMIC), located at the VA Iowa Healthcare System (Iowa City, IA), an internal VA transcription service, to transcribe the digitally recorded semi-structured provider interviews (Study 1). The investigator team executed a data use agreement (**DUA)** with the service (Appendix 1). They will receive the recorded interviews and return verbatim transcriptions to the investigators as described in Study Procedures. All study data sent between EMIC and VA Boston will be transferred via secure file transfer behind the VA firewall. Handbook 6500.6 establishes VA's procedures, responsibilities, and processes for implementing security in appropriate contracts and acquisitions in which VA sensitive information is stored, generated, transmitted or exchanged by VA and a contractor regardless of format and whether it resides on a VA or a non-VA system. In the event that EMIC does not have capacity, we will engage another qualified transcription provider and execute the required data use agreement as we have done in the past.

5.0 Study Procedures

5.1 Study Design

Study 1 (Providers)

A. Experimental Design

Research qualitative assessments of provider perceptions of care will be conducted in the context of the OMHO-sponsored randomized program evaluation described above. Utilizing a stepped wedge design[50,58-60], REP-F implementation support will be provided to 9 volunteer VAMCs in three waves of three sites each. Providers will be consented to undergo semi-structured interview prior to and after 12 months of REP-F support, consenting for both interviews at enrollment. Interview data will be analyzed qualitatively; utilizing both directed content analysis to identify CCM elements in use as well as grounded thematic analysis to identify implementation barriers and facilitators.

Four BHIP providers will be recruited at each of 9 VAMCs receiving REP-F support, using recruitment techniques from our prior project, QUERI RRP #13-237/VA CIRB#13-48. We will work with VAMC Mental Health leadership to identify all providers working within the local BHIP team. From that list we will select four providers per site, including at least one physician, psychologist, registered nurse, advanced practice nurse, medical support assistant, peer support specialist and social worker in each group. Note that VAMC Mental Health leadership will not be informed of which particular providers we selected, or whether any participants refused or agreed to the interview. The interviewer and assistant will take field notes after each interview, noting key issues that arose, immediate impressions of the interviewee's perspectives on BHIP implementation and what impact BHIP care appears to have on patients and providers. We will audio record these interviews and they will be transcribed by EMIC, located at VA Iowa City Healthcare System, and its contracted replacement. We will reconcile transcripts and interview notes and code interviews using a rapid assessment procedure, an anthropological approach to assessing real-time processes and procedures to rapidly inform policy development [55]. Coding will focus on: discriminating presence or absence of each of the six CCM components in BHIP care.

B. Minimization of Risk

See (D) below.

C. Study Population

The study population will consist of four BHIP providers at each of 9 participating VAMCs. The names of eligible providers (the population) will be supplied by the VAMC and the sampling and consenting will be done by investigators from VABHS.

Four BHIP providers from each of 9 sites, including at least one physician, psychologist, registered nurse, advanced practice nurse, medical support assistant, peer support specialist and social worker (for a total of 36 providers) will be needed. Each will be interviewed prior to and after 12 months of REP-F support. If an individual consented at baseline (the initial interview) declines to participate at 12 months, or has left VA employment, another BHIP provider will be consented and interviewed.

D. Added Protections for Vulnerable Populations

This research will be conducted with employees, a vulnerable population with which we are quite experienced. For the telephone interview with providers, we will utilize the Central IRB-approved study fact sheet with the elements of informed consent which details potential risks and precautions taken to guard against the risks. Recognizing that employees are vulnerable subjects, the consent process will emphasize that the interviews are voluntary and confidential and that their participation or non-participation will not be reported to the employee subjects' supervisors or managers. *For instance, even if we do not meet recruitment goals, we will not disclose this information to supervisors and managers since it could imply noncooperation by some or all of the employees potentially involved*. Importantly, no individual level data will be reported from any source; all data will be reported in aggregate form to ensure that any findings from the study cannot be traced back to any specific provider. That is, not only will names not be disclosed; we will not disclose sufficient information to trace back data deductively to its source. *For instance, if a transcript reveals that a social worker said, "I cannot get sufficient medication support from the psychiatrist to do good care management" those data would remain confidential with the investigator team; for scientific presentations, publications, or other reports such data might be reworded, "I cannot get sufficient support from the other disciplines to do my job effectively."* We have utilized such redaction methods in our previous work [53].

E. Data Storage

There will be no specimens or specimen banking.

All information on individual subjects will be kept confidential on restricted-access server files behind the VA firewall. Access to the analytic files within VA will be limited to the principal investigator and those team members involved in the analyses. Individually unique user identification codes and passwords are necessary to access the network. Accounts and passwords comply with existing VA policy and procedures.

Digital recordings of the telephone provider interviews will be downloaded to secure restricted-access server files behind the VA firewall following the interviews and then immediately deleted from the devices.

Hard copies of the provider interview transcripts will be kept in a locked area of research space at engaged VAMCs (VABHS, CAVHS). Hard copies will be maintained in accordance with the VA Record retention schedule:
www1.va.gov/VHAPUBLICATIONS/RCS10/rcs10-1.pdf

Only research project staff with credentials and permission to use the data will have access to the data.

Study 2 (Veterans)

A. Experimental Design

One of the innovative aspects of this multi-site study is that we will take a population-level approach to assessing health status and perceptions of care. Thus, at each of the nine sites in which we are delivering our REP-F facilitation intervention at the provider level, we will also conduct telephone-based quantitative interviews with Veterans receiving care through the local BHIP team. We will administer these interviews to 85 Veterans at each of three time points at each site: at the start of our facilitation, midway through facilitation (Month 6), and at the end of facilitation (Month 12).

Similar to our procedures in QUERI RRP 13-237/VA CIRB 13-48, Mental Health Service administration at each of the nine sites will provide investigators with the clinic names for clinicians who have treated Veterans in BHIP clinics over the past year. We will request a HIPAA Waiver of Authorization (attached) to access Veteran data since the conduct of this research would not be practical or valid if individual subject contacts were necessary. From this, we will identify all Veterans who received 2 or more outpatient encounters in that clinic in the prior year; from this point forward, Veterans will be identified by scrambled SSN (**SCRSSN**) rather than SSN or name. We will gather the following data on these Veterans via the CDW: demographics (age, gender, minority status, marital status, disability), psychiatric diagnoses in the past year, cardiometabolic diagnoses (hypertension, hyperlipidemia, obesity, diabetes mellitus), service utilization (inpatient days, outpatient visits), and medication usage.

We will exclude those with an encounter for a diagnosis of dementia (ICD-9 code 290.xx, 294.1, 331.0). We will then mail-out a study information packet including an opt-out postcard (details below). We will then submit a look-up file of the SCRSSNs of those Veterans to staff at the CDW, and they will provide name and mailing address information for them so that packages can be mailed. Each of these potentially eligible Veterans will be mailed the study information package consisting of (1) a study information fact sheet from a member of our research team; and (2) an opt-out postcard and (3) an invitation letter (see Appendix 4). The recruitment letters will include a callback number for research staff and will state that, if we do not receive a phone call or the opt-out postcard within two weeks from the date of the mailing, a research assistant may call the Veteran to explain the study in more detail. The opt-out postcard strategy has been used in other research studies and has been highly successful (e.g. it obtained approximately a 25% participation rate for phone-based Veteran interviews in Dr. Jeffrey Pyne's CREATE 12-300/VA CIRB 13-29).

Verbal consent will be obtained during the initial phone call. We will request a Waiver or Alteration of Informed Consent (attached) to allow for verbal rather than written consent procedures.

For those who meet eligibility criteria and agree to participate in the study, the research assistant will then verbally administer (over the telephone) a battery that includes self-reported race/ethnicity and employment status, the Patient Assessment of Chronic Illness Care (**PACIC**)[61], the Quality of Life, Enjoyment, and Satisfaction Questionnaire (**QLESQ**)[62], the Satisfaction Index[63], and the Veterans Rand 12-item Social Functioning measure (**VR-12**)**[64],** found in Appendix 5. This includes a total of 51 items. The Veteran will be given the opportunity to have the health status measures (VR-12, Q-LES-Q) communicated to the VA

provider of their choice.   Communication with the provider will be done via encrypted email from the research assistant within one business day (see email text, Appendix 6)

Our recruitment goal is to administer telephone-based interviews to 85 Veterans at the start of the REP-F facilitation implementation (Month 0) at each site, and to re-contact these same Veterans for follow-up interviews using the same measures at Month 6 and Month 12. We anticipate approximately 15% dropout at each of these time points, and will replace such dropouts from our original pool of Veterans who were sent the study information package but who did not opt out of the study. The overall goal is to therefore administer telephone-based interviews to 85 Veterans at each of these three time points, some of whom will have been interviewed three times, some twice, and some once. Consent script will reflect this heterogeneity, asking permission to be interviewed "up to three times in the next 12 months," and will be adjusted accordingly for those who are interviewed for the first time at 6 months ("up to twice in the next six months") or at 12 months (one-time interview). Our generalized linear modeling statistical approach can manage this heterogeneity in the sample (see below).

Note that we also provide the Veteran with the option to communicate his/her health status to the provider of their choice, to support the use of measurement-based care. This will be done via email using the template in Appendix 6. Note that this process is independent from the harm risk screening and mitigation procedures described in the next section.

B. Minimization of Risk

Personal identifiers (names and mailing addresses) will be collected and kept until the VA Record retention schedule allows their destruction. Data from the telephone-based interview will be given a unique numeric code; the coding crosswalk will be available only to the VA Boston research study team.

As above, eligible Veterans will receive the study information package including a description of the study, and an opt-out response postcard. The study description will emphasize that participation is entirely voluntary, and Veterans may opt out of any further contact from the study team by calling the included phone number or returning the opt-out response postcard.

Once the study research assistant reaches the Veteran by telephone, the research assistant will review the elements of informed consent (see Appendix 5 for interview script). The research assistant will also emphasize that completion of the telephone interview is entirely voluntary, and the Veteran may decline to complete it or may skip items for any reason.

Note that the telephone-based interview items, mainly drawn from our RRP protocol, are from instruments that have been published and widely used as health status or healthcare process self-report assessments, or are being used by VA operational assessments. None are probing, uncovering, or projective questions that would tend to cause emotional upset.

Nonetheless, we have taken extreme care to put in place a process to respond to potential harm risk that may be disclosed during the telephone interview. This harm risk procedure follows the protocol utilized by OMHO in their Veterans Outcome Assessment, which randomly selects Veterans newly enrolled in mental health clinics across the country and assesses their health status and satisfaction with care in a longitudinal evaluation process. Moreover, we have substantive support from the OMHO Veterans Crisis Line, who will provide 24/7 access for our interviewers in the event that a Veteran discloses harm risk (see Weaver letter Appendix 7).

Specifically, as with the OMHO Veterans Outcome Assessment protocol, each Veteran will be screened during the interview of current thoughts of harm to self using the suicidality item from the PHQ-9, a depression measure widely used in VA clinical care. Those who screen positive for any thoughts of harm to self will then be assessed with P4 suicidality screener[65]. The P4 stratifies respondents into three risk strata, each with a corresponding risk response:

- Minimal Risk
- Lower Risk
- Higher Risk

Veterans who screen into any of these risk categories will be offered access to the Veterans Crisis Line (800-273-TALK), and be encouraged to contact their provider. The research assistant will also contact the study clinician (Dr. Bauer, Dr. Miller, or Dr. Kirchner) who will send an encrypted email notification to the Suicide Prevention Coordinator at their VA medical center detailing the risk stratification and relevant interview notes regarding response to harm risk questions. Email notification will be sent within one business day. As part of Veterans Crisis

Line support, they are also providing our interviewers with a special internal phone number and rapid-response email group for situations in which a Veteran requires warm handoff. For less acute follow-ups, as with the OMHO survey, we choose to contact the Suicide Prevention Coordinator rather than the Veteran's provider because the Veteran may be cared for by multiple providers, or may not know who to contact, or may refuse to give a provider's name, and the Suicide Prevention Coordinator is in the best position to identify and contact the appropriate provider locally. Additionally, if the research assistant is in any way concerned about harm risk in the moment, and the Veteran refuses warm hand-off to the Veterans Crisis Line, they themselves will contact the Veterans Crisis Line and provide the name and contact information for the Veteran. This will also be communicated via email from the study clinician to the Suicide Prevention Coordinator within one business day. The risk stratification and response scheme and relevant email texts are found in Appendices 5 and 6.

If the Veteran refuses to answer any harm risk questions, the study clinician may conduct an initial assessment of likelihood of harm with the Veteran to determine if contacting the Suicide Prevention Coordinator and/or the Veterans Crisis Line on the Veteran's behalf is necessary to prevent harm to the Veteran or others.

Each potential respondent will have a unique personal identifier. The linking file and personal identifying information will be known only to study personnel at VABHS and CAVHS involved with the data preparation and analyses on a need-to-know basis.

C. Study Population

The study population consists of Veterans who have had 2 or more outpatient encounters with a BHIP team clinician at the nine participating sites within the prior year. We exclude those with an encounter for a diagnosis of dementia in the prior year, as outlined above. Our selection of participants for the telephone-based interview will result in a sample that mirrors the gender and racial distribution of the Veteran population treated within those BHIP teams.

D. Added Protections for Vulnerable Populations

We are sampling Veterans by identifying those who have seen specific providers—i.e., providers practicing in BHIP teams. However, since we are taking a population-based approach, we are interested in population-level analyses. We thus will not analyze data in a manner that could provide profiles of, e.g., health status or medication usage that could be tracked back to an identified Veteran.

E. Data Storage

All information on individual subjects will be kept confidential on restricted-access server files behind the VA firewall at VABHS and CAVHS. Veteran interview data will be entered directly into and stored in the VA's Research Electronic Data Capture (**REDCap**) system at CAVHS within the VA firewall (no hard copies of interview data will be made). Access to the analytic files within VA will be limited to the principal investigator and those team members involved in the analyses. Individually unique user identification codes and passwords are necessary to

access the network. Accounts and passwords comply with existing VA policy and procedures. Access to the CDW databases requires authorization by VA national data systems which the study data analysts have received.

Hard copies of the provider interviews (Study 1) will be kept in a locked area of CHOIR space at VABHS. No hard copies of Veteran interview data will exist since data will be entered directly into REDCap; opt-out postcard responses will be kept in a locked area of CHOIR space at VABHS. Hard copies will be maintained in accordance with the VA Record retention schedule: www1.va.gov/VHAPUBLICATIONS/RCS10/rcs10-1.pdf

Only research project staff with credentials and permission to use the data will have access to the data.

All data entry will take place behind the VA firewall, by research team members. The data files will be stored in restricted-access, secure server drive restricted for project-specific work. All data analyses for Study 1 and Study 2 will also take place on restricted-access, secure server drives restricted for project-specific work.

5.2 Recruitment Methods

Study 1 (Providers)

A. State how many subjects will be needed.

Four BHIP providers from each of 9 sites, including at least one physician, psychologist, registered nurse, advanced practice nurse, medical support assistant, peer support specialist and social worker (for a total of 36 providers) will be needed. Each will be interviewed prior to and after 12 months of REP-F support. If an individual consented at baseline (the initial interview) declines to participate at 12 months, or has left VA employment, another BHIP provider will be consented and interviewed.

B. Describe when, where, how and by whom potential subjects will be identified and recruited.

Each VAMC's Mental Health Service will provide the names of eligible providers to the investigators and will send an email introducing the study (attached) to eligible providers. Investigators will then randomly order the list of eligible providers according to discipline and invitations will be made via email in order until the desired sample size is reached. Each provider will receive an initial invitation and one follow-up email. If insufficient responses are available in a given discipline, we will recruit other eligible discipline members to achieve the desired sample size of four providers per VAMC. No follow-up urging or communication regarding recruitment will be requested from Mental Health Service after the first email of introduction in order to reduce the potential for coercion or assessment of participation by an employee's supervisor.

C. Describe materials that will be used to recruit subjects, e.g., advertisements. Include materials as an appendix or separate attachment.

Two emails will be sent to providers who may be eligible to participate in the study (attached). The first, from the Mental Health Service, will describe the study to potential participants but is for informational (rather than recruitment) purposes ONLY.

The second email, from the study investigators, will be used to recruit potential participants and will only be sent after the mental health service chief has sent the first email. Each provider will receive an initial invitation and a follow-up email (up to three times) and follow-up by telephone (up to two times) to determine willingness to participate.

In addition, after recruitment, we will send participants a thank you certificate with an overview of preliminary results of the study to express our gratitude for their time and acknowledge their interest in study outcomes. This certificate is attached (Appendix 6).

D. Describe any payments to subjects

None.

<u>Study 2</u> (Veterans)

A. State how many subjects will be needed.

Eighty-five Veterans treated by BHIP teams at each of the nine sites for each of three study time-points will be needed. As outlined above, we will recruit a sufficient number of Veterans to reach this number at each time point at each VA medical center.

B. Describe when, where, how and by whom potential subjects will be identified and recruited.

See Section 5.1 (Study2) A. above.

C. Describe materials that will be used to recruit subjects, e.g., advertisements. Include materials as an appendix or separate attachment.

The study information package is attached (Appendix 4), as is the telephone interview script to be used by the research assistant for telephone follow-up (Appendix 5). No additional recruitment materials will be utilized.

In addition, after recruitment, we will send participants a thank you certificate with some preliminary results of the study to express our gratitude for their time and acknowledge their interest in study outcomes. This certificate is attached (Appendix 6).

D. Describe any payments to subjects

None.

Note on Engagement in the Conduct of Research
We consider BHIP sites not to be engaged in the conduct of research, as described in the U.S. Department of Health and Human Services, OHRP, *Guidelines on Engagement of Institutions in Human Subjects Research*, particularly Section III.B (http://www.hhs.gov/ohrp/policy/engage08.html)

Sites whose roles are limited to providing subjects for this research project are considered not engaged in research (though of course VABHS and CAVHS are engaged in research by virtue of their investigators on the study). Specifically, the 9 participating VAMCs will provide information about the study to the population of eligible providers. They will also release information to the investigators on potential subjects, including identifiable information; however, the provision of such information is consistent with VAMC operational (non-research) duties, such as they would do for External Peer Review, OMHO reviews, and other internal business or clinical operational tasks. Their staff will not conduct interviews, provide interventions, or consent subjects. Furthermore, their role in providing information will not merit professional recognition or publication authorship of study results. We will notify facility leadership before we arrive on-site to engage in any recruitment activities or conduct the interviews.

5.3 Informed Consent Procedures

Study 1 (Providers)

We will interview 4 providers at each of the 9 BHIP sites for a total of 36 providers. Dr. Sullivan and Dr. Drummond will conduct the interviews by telephone which will be audio recorded. The providers will be interviewed 2 times over the course of the study: one time before the facilitation process and a second time 12 months later. The investigators obtaining consent are from VABHS and are health services researchers experienced at obtaining informed consent. We will consent the providers for both interviews in a single consent prior to the first interview. If an individual consented at baseline (the initial interview) declines to participate at 12 months, or has left VA employment, another BHIP provider will be consented and interviewed.

For the telephone interview with providers, we will utilize the Central IRB-approved study fact sheet with the elements of informed consent which details potential risks and precautions taken to guard against the risks. The study fact sheet will be included with the provider interview invitation. Recognizing that employees are vulnerable subjects, the consent process will emphasize that the interviews are voluntary and confidential and that their participation or non-participation will not be reported to the employee subjects' supervisors or managers nor will the content of the interview be reported. While documentation of informed consent is not required for telephone interviews, at the start of the interview, we will review the study fact sheet

elements of consent and ask the provider if he/she verbally consents to conducting the interview and to recording the interview. If the provider consents to audio recording, we will repeat the verbal consent so that it is captured on the recording. A note taker will assist at the interviews should a provider be willing to conduct an interview but would prefer that no audio recording be made.

Study 2 (Veterans)

We are seeking a Waiver or Alteration of the Informed Consent process as well as a Waiver of Documentation of Informed Consent for the telephone-based interview. For the telephone interview with Veterans, we will utilize the Central IRB-approved study fact sheet with the elements of informed consent which details potential risks and precautions taken to guard against the risks. The study fact sheet will be included with the Veteran interview invitation. Veterans will also receive an opt-out postcard to return if they do not wish to participate in the telephone interviews. If Veterans choose not to return the opt-out postcard, they will receive a telephone call asking them to participate in the telephone survey. The Veterans will have 2 opportunities to consent or not consent to participation: the return of the opt-out postcard and at the start of the telephone interview. As part of the informed consent process at the start of the interview, we will review the elements of consent and answer any questions the participant may have about participation. By answering the interview questions, the Veterans are consenting to participate in the study.

5.4 Inclusion/Exclusion Criteria

Study 1 (Providers)

Inclusion criteria: Employees must be Mental Health staff who are members of BHIP teams at participating sites.

Exclusion criteria: Within the broader population of BHIP staff as defined by the inclusion criterion above, there will be no exclusion criteria.

Study 2 (Veterans)

Inclusion criteria to receive the study information package: to be eligible to participate, Veterans must have had 2 or more encounters with a BHIP team clinician at one of the nine sites in the prior year and be at least 18 years of age.

Exclusion criteria to receive the study information package: Veterans with an encounter for dementia within the prior year will be excluded from participation.

5.5 Study Evaluations

Study 1 (Providers)

The provider interview is attached. This is a semi-structured interview adapted from one of our prior CCM studies [53], designed to assess the concordance of care for returning Veterans with CCM principles. We have adapted the interview for use in the BHIP initiative with a particular focus on (1) eliciting CCM components that are vs. are not present in GMH care along with an assessment of existing system redesign processes and (2) implementation barriers and facilitators based on the work of Powell [57] and Kirchner [45]. The interview lasts approximately45-45-60 minutes. The interviewer and assistant will take notes during the interview, and the interview will be audio recorded and transcribed verbatim.

Study 2 (Veterans)

The text of the telephone-based interview is attached (Appendix 5). It will include verbal administration of the PACIC, Q-LES-Q, Satisfaction Index and VR-12, plus self-reported race/ethnicity and current employment status.

5.6 Data Analysis

Study 1 (Providers)

The sample size of four providers per VAMC each interviewed twice (72 total interviews, across 36 distinct providers) is based on prior developmental qualitative experience and should suffice to illustrate the themes and variability that will emerge. Data will be analyzed by investigators working behind the VA firewall on password-protected servers at VABHS and CAVHS.

Transcripts, augmented with interviewer notes, will be reconciled and subjected to qualitative analyses (directed content analysis and grounded thematic analysis), aided by NVivo qualitative data management software or equivalent. We will code interviews using a rapid assessment procedure, an anthropological approach to assessing real-time processes and procedures to rapidly inform policy development [55]. Coding will focus on: (1) discriminating presence or absence of each of the six CCM components in “typical” GMH care, and (2) perceived and desired implementation support[57]; in this we will also particularly attend to attitudes and capabilities regarding using informatics to communicate with patients and other providers. Regarding the presence versus absence of the six CCM components, we expect the rank order of evaluations to indicate that post-REP-F care is more consistent with CCM elements than pre-REP-F care.

Data from qualitative analyses will serve two purposes. First, directed content analysis[56] focusing on identification of CCM elements will provide data with which to assess fidelity to the intervention dependent variable for our research hypothesis. Second, grounded thematic analysis[54] will contribute to the implementation science literature regarding barriers and facilitators of implementation.

To conduct the **directed content analysis**[56] to identify CCM elements, Dr. Sullivan will identify quotes relevant to the presence or absence of each of the six CCM elements. Quotes will be deidentified and information regarding the provider, site, and pre/post implementation status of each quote will be kept confidentially by Dr. Sullivan. After the final interview is conducted, deidentified quotes will be consolidated into a "quote pool" and each quote will be coded by Drs. Drummond, Sullivan, Elwy, Kim, and Miller dichotomously as to the presence/absence of a CCM element in the quote, using the codebook developed in the RRP. Coding will be done without knowledge of provider, site, or time of interview. Quote ratings (CCM element = present/absent) will then be reassembled into a coding sheet for each interview, organized according to each of the six CCM elements. If no quotes support the presence of a particular CCM element, it will be coded as absent. Each of the 72 interviews will then be summarized with regard to which CCM elements were endorsed as present during the interview, and an overall score (0-6 for the number of CCM elements endorsed as present) will be assigned for each interview. These CCM scores for each of the 72 interviews (4 providers/site, 9 sites, pre- and post-implementation) will then be rolled up to the site level, taking the mean interview CCM score as the overall site score. These 18 site-level scores (two scores each for each of the 9 sites, one pre-implementation and one post-implementation) will serve as the basis for analyses.

Formal statistical analyses of directed content analysis data are not appropriate[56]. However, our *a priori* hypothesis is that for each site CCM scores will increase from pre- to post-implementation, and we will be able to describe the degree to which this occurred within and across sites. Additionally, we will assess individual elements from provider's ratings in each site to identify patterns of implementation across sites for individual CCM elements, which would add internal consistency and validity to our conclusions; that is: common patterns would support (though not prove[56]) generalizability of implementation strategy effects.

To conduct the **grounded thematic analysis**, Drs. Drummond, Sullivan, Elwy, Miller, and Kim will separately code interviews using previously developed grounded thematic analysis[54] codes, paying particular attention to newly emergent codes and factors that might be barriers or facilitators to future implementation efforts[57]. These analyses will also be used to contextualize and explain our direct content analyses above.

Study 2 (Veterans)

For the three administrations of the telephone-based Veteran interview at each site, repeated measures general linear model analyses (**GLM**)[81-83] will control for site characteristics (between-site effects) and calendar time, which reflects secular trends (within-site effects, as well as the number of interview assessments each Veteran contributes. Based on Parchman's stepped wedge CCM trial,[73] discussions with Dr. Parchman, our experience with QUERI RRP 13-237/VA CIRB 13-48, and OMHO's operational experience, we anticipate that maximal effects will not be seen until the end of the step-down period, i.e., after 12 months of implementation support; however, our approach also allows us to detect other patterns, e.g., prompt response at 6 months and fall-off by 12 months.

GLM quantifies and apportions the variance in outcome (e.g., VR-12 subscale scores) among relevant factors, thus isolating the change in outcome due to the primary contrast of interest (in

this study, implementation support vs. waiting). GLM accommodates repeated measures (within-subject correlation), random effects (subject), moderate imbalance among independent factors (sites), and various types of missing data, as well as replacement of subjects who are not represented at each of the three study time points.[84,85] GLM also allows us to explore results for patterns of unequal variance, relevant correlation structures, and variance component models to ensure that our results are robust. Additionally, the site sample sizes are large enough to explore many site-specific effects by adding site-interaction terms to the model.

5.7 Withdrawal of Subjects

Study 1 (Providers)

Provider subjects may withdraw from the study at any time by verbal request, which will be documented in the case file by the investigator. Withdrawal will not be communicated to any site staff in order to maintain confidentiality and avoid the potential for coercion.

Study 2 (Veterans)

As above, the study information package (mailed to eligible Veterans) will include an opt-out postcard as well as a phone number for Veterans to call if they do not wish to participate. Furthermore, once contacted by phone by the study research assistant, Veterans will once again have the opportunity to withdraw by simply verbally declining to participate at any time.

6.0 Reporting

Dr. Bauer will lead regular study investigator meetings and will formally query the investigator team specifically for any serious adverse events, unanticipated occurrences related to subject safety, breaches of study protocol or confidentiality no less than quarterly, as he has done for past studies (IIR #10-314 and RRP#13-237/VA CIRB 13-48). Study investigators will also be directed to bring any such problems to the attention of the PI as they occur. The PI will report relevant occurrences both to the Central IRB and to the site R&D Committees per VA procedures.

7.0 Privacy and Confidentiality

See also Section 4.D, “Added Protection for Vulnerable Populations.”

Investigators will not disclose subjects’ PII (Study 1) or PHI (Study 2). All investigators and staff engaged in research will have complied with all VA research training requirements prior to beginning participation in this study.

All information on individual subjects will be kept confidential on restricted-access server files behind the VA firewall. Study staff will obtain a list of the names and addresses of BHIP staff from mental health leadership at each site in order to recruit providers to complete telephone interviews. The study PI and Qualitative Lead will maintain an electronic file linking identification numbers to the names of the provider interview participants (Study 1 “crosswalk”), with a similar file for Veterans maintained by the study PI and Study 2 staff, Ms. Stolzmann, Ms. Riendeau, and Mr. Pitcock. These files will be in a folder that is separate from other research data. Access to the analytic files within VA will be limited to the principal investigator and those team members involved in the analyses. Individually unique user identification codes and passwords are necessary to access the network. Accounts and passwords comply with existing VA policy and procedures.

Hard copies of subject responses (Study 1 provider interviews; Study 2 opt-out postcards) will be kept in a locked area of CHOIR space at VA Boston Healthcare System. Hard copies will be maintained in accordance with the VA Record retention schedule (www1.va.gov/VHAPUBLICATIONS/RCS10/rcs10-1.pdf). Only research project staff with credentials and permission to use the data will have access to the data.

Involvement of Outside Vendors

There are no outside vendors contracted for this study at this time.

8.0 Communication Plan

All site approvals (VABHS, CAVHS, VAWNYHCS and PVAMC) will be obtained in compliance with Central IRB and local R&D procedures. For each site participating but not engaged in research (in this study, the 9 sites receiving BHIP implementation facilitation), we will notify the Director prior to conducting research at his/her facility. Also we will process any changes in the protocol, informed consent process, or cessation of engagement in research for any sites in concordance with Central IRB and local site procedures.

The PI will meet regularly with the investigator team, which includes the Local Site Investigators from CAVHS (Dr. Kirchner), VAWNYHCS (Dr. Wray) and PVAMC (Dr. Oslin) and through this mechanism we will facilitate all above communications. This process has worked well with the Central IRB-approved IIR #10-314 and RRP#13-237.

9.0 References

1. Kessler RC, Wang PS. The descriptive epidemiology of commonly occurring mental disorders in the United States. Ann Rev Public Health 2008; 29:115-129.
2. Hogan MF: New Freedom Commission Report: The President's New Freedom Commission: Recommendations to transform mental health care in America. Psychiatr Serv 2003; 54(11):1467-74.
3. Watkins, K. E., H. A. Pincus, Paddock S, Smith B, Woodroffe A, Farmer C, Sorbero ME, Horvitz-Lennon M, Mannle T, Hepner KA, Solomon J, Call C. Care for veterans with mental and substance use disorders: good performance, but room to improve on many measures. Health Aff 2011; 30(11): 2194-2203.
4. VHA. Uniform Mental Health Services Handbook. *VHA Handbook 1160.010*. June 11, 2008.
5. US Senate Committee on Veterans Affairs. On-line transcript of Senate hearing: “VA Mental Health Care: Addressing Wait Times and Access to Care”, 11/30/2011. Accessed 8/21/14.
6. VA Office of the Inspector General. Statement before the Committee on Veterans Affairs of the US House of Representatives Hearing on VA Mental Health Care Staffing; 5/8/2012. Accessed 8/21/14.
7. Kitson AL, Rycroft-Malone J, Harvey G, McCormack B, Seers K, Titchen A. Evaluating the successful implementation of evidence into practice using the PARiHS framework: theoretical and practical challenges. Implement Sci 2008;3:1.
8. Stetler CB, Damschroder LJ, Helfrich CD, Hagedorn HJ. A Guide for applying a revised version of the PARIHS framework for implementation. Implement Sci 2011; 6:99.
9. Wagner EH, Austin BT, Von Korff M. Organizing care for patients with chronic illness. Milbank Q 1996; 74(4):511-544.
10. Von Korff M, Gruman J, Schaefer J, Curry SJ, Wagner EH. Collaborative management of chronic illness. Ann Intern Med 1997; 127(12):1097-102.
11. Improving Chronic Illness Care Program, Group Health Research Institute. Web-based overview of CCM. Accessed 8/21/14.
12. Bodenheimer T, Wagner EH, Grumbach K. Improving primary care for patients with chronic illness: The chronic care model, part 1. JAMA 2002; 288 (14):1775-1779.
13. Bodenheimer T, Wagner EH, Grumbach K. Improving primary care for patients with chronic illness: The chronic care model, part 2. JAMA 2002; 288(15):1909-1914.
14. Coleman K, Austin BT, Brach C, Wagner EH. Evidence on the chronic care model in the new millennium. Health Aff 2009; 28(1):75-85.
15. Badamgarav E, Weingarten SR, Henning JM,Knight K, Hasselblad V, Gano Jr. A, Ofman JJ. Effectiveness of disease management programs in depression. Am J Psychiatry 2003; 160(12):2080-2090.
16. Gilbody S, Bower P, Fletcher J, Richards D, Sutton AJ. Collaborative Care for Depression: A Cumulative Meta-Analysis and Review of Longer-term Outcomes. Arch Intern Med 2006; 166:2314-2321.
17. Rubenstein LV, Chaney EF, Ober S, Felker B, Sherman SE, Lanto A, Vivell S. Using evidence-based quality improvement methods for translating depression collaborative care research into practice. Fam Syst Health 2010 Jun;28(2):91-113.
18. Bauer MS, McBride L, Williford WO, Glick HA, Kinosian B, Altshuler L, Beresford TP, Kilbourne AM, for the CSP #430 Study Team. Collaborative care for bipolar disorder, Parts I&II: intervention and implementation in a randomized effectiveness trial. Psychiatr Serv 2006; 57:927-36 & 937-45.

19. Simon GE, Ludman EJ, Bauer MS, Unützer J,Operskalski B. Long-term effectiveness and cost of a systematic care management program for bipolar disorder. Arch Gen Psychiatry 2006; 63:500-08.
20. Kilbourne AM, Post EP, Drill LJ, Cooley S, Bauer MS. Improving medical and psychiatric outcomes for individuals with bipolar disorder: a randomized controlled trial. Psychiatr Serv 2008; 59:760-68.
21. Kilbourne AM, Goodrich DE, Lai Z, Post EP, Schumacher K, Nord KM, Bramlet M, Chermack S, Bauer MS: Randomized controlled trial to reduce cardiovascular disease risk for patients with bipolar disorders: the Self-Management Addressing Heart Risk Trial (SMAHRT). J Clin Psychiatry 2013; in press.
22. Kilbourne AM, Goodrich D, Lai Z, Clogston J, Bauer MS, Waxmonsky J. Randomized controlled pilot study of Life Goals Collaborative Care for patients with bipolar disorder and cardiovascular disease risk. Psychiatr Serv 2012; 63:1234-38.
23. Kilbourne AM, Li D, Lai Z, Waxmonsky J, Ketter T. Pilot randomized trial of a cross-diagnosis collaborative care program for patients with mood disorders. Depr & Anx 2013; 30:116-32.
24. Yatham LN, Kennedy SH, O'Donovan C, Parikh SV, MacQueen G, McIntyre RS, Sharma V, Beaulieu S: Canadian Network for Mood and Anxiety Treatments (CANMAT) guidelines for the management of patients with bipolar disorder: Update 2007. Bipolar Disord 2006; 8(6):721-739.
25. Department of Veterans Affairs & Department of Defense. Clinical practice guideline for management of bipolar disorder in adults, version 2.0. Department of Veterans Affairs Office of Quality and Performance & US Army MEDCOM Quality Management Division. 2009.
26. USDHHS Substance Abuse and Mental Health Administration National Registry of Evidence-Based Programs and Practices (NREPP). Web-based search engine for NREPP-listed programs. Accessed 8/21/14.
27. Woltmann E, Grogan-Kaylor A, Perron B, Georges H, Kilbourne AM, Bauer MS. Comparative effectiveness of collaborative chronic care models for mental health conditions across primary, specialty, and behavioral health settings. systematic review and meta-analysis. Am J Psychiatry 2012; 169:790-804.
28. Miller CJ, Grogan Kaylor A, Perron BP, Woltmann E, Kilbourne AM, Bauer MS. Collaborative chronic care models for mental health conditions: cumulative meta-analysis and meta-regression to guide future research and implementation; Medical Care 2013; 51:922-30.
29. Coleman EA, Grothaus LC, Sandhu N, Wagner EH. Chronic care clinics: a randomized controlled trial of a new model of primary care for frail older adults. J Ame Geriatr Soc 1999; 47:775-85.
30. Druss BG, Rohrbaugh RM, Levinson CM, Rosenheck RA. Integrated medical care for pateints with serious psychiatric illness: a randomized trial. Arch Gen Psychiatry 2001; 58:861-68.
31. Oslin DW, Sayers S, Ross J, Kane V, Ten Have T, Conigliaro J, Cornelisu J. Disease management for depression and at-risk drinking via telephone in an older population of veterans. Psychosom Med 2003: 69:931-37.
32. Smith RC, Lyles JS, Gardiner JC, Sirbu C, Hodges A, Collins C, Dwamena FC, Lein C, William Given C, Given B, Goddeerus H. Primary care clinicians treat patients with medically unexplained symptoms: a randomized controlled trial. J Gen Intern Med 2006; 21:671-77.
33. Van Stratten A, Tiemens B, Hakkaart L, Nolen WA, Donker MC. Stepped care vs matched care for mood and anxiety disorders: a randomized trial in routine practice. Acta Psychiatr Scand 2006; 113:468-76.
34. van Orden M, Hoffman T, Haffmans J, Spinhoven P, Hoencamp E. Collaborative mental health care versus care as usual in a primary care setting. Psychiatry Serv 2009; 60:74-79.

35. Druss BG, von Esenwein SA, Compton MT, Rask KJ, Zhao L, Parker RM. A randomized trial of medical management for community mental health settings. Amer J Psychiatry 210; 167:151-59.
36. Patel V, Weiss HA, Chowdhary N, Naik S, Pednekar S, Chatterjee S, DeSilva MJ, Bhat B, ARaya R, King M, Simon G, Verdeli H, Kirkwood BR: effectiveness of an intervention led by lay health counselors for depressive and anxiety disorders in primary care in Goa, India (MANAS). Lancet 2010; 376:2086-95.
37. Zatzick D, Roy-Byrne P, Russo J, Rivara F, Droesch R, Wagner A, Dunn C , Jurkovich G, Uehara E, Katon W: A randomized effectivenesstrial of stepped collaborative care for acutely injured trauma survivors. Arch Gen Psychiatry 2004; 61:498–506.
38. Fortney JC, Pyne JM, Kimbrell TA, Hudson TJ, Robinson DE, Schneider R, Moore WM, Custer PJ, Grubbs K, Schnurr PP. Telemedicine-based collaborative care for posttraumatic stress disorder. a randomized clinical trial. JAMA Psychiatry 2014. doi:10.1001/jamapsychiatry.2014.1575.
39. Schnurr PP, Friedman MJ, Oxman TE, Dietrich AJ, Smith MW, Shiner B, Forshay E, Gui J, Thurston V. RESPECT-PTSD: re-engineering systems for the primary care treatment of PTSD, a randomized controlled trial. J Gen Intern Med 2012; 38:32-40.
40. Saitz R, Cheng DM, Winter M, Kim TW, Meli SM, Allensworth-DAvies D, Lloyd-Travaglini CA, Samet JH. Chronic care management for dependence on alcohol and other drugs. JAMA 2013; 310:1156-67
41. Neumann MS, Sogolow ED. Replicating effective programs: HIV/AIDS prevention technology transfer. AIDS Educ Prev 2000;12(5 Suppl):35-48.
42. Kilbourne AM, Neumann MS, Pincus HA, Bauer MS, Stall R. Implementing evidence-based interventions in health care: application of the Replicating Effective Programs framework. Implement Sci 2007; 2:42
43. Kilbourne AM, Neuman MS, Waxmonsky J, Bauer MS, Kim HM, Pincus HA, Thomas M. Evidence-based implementation: The role of sustained community-based practice and research partnerships. Psychiatr Serv 2012; 63:205–207, 2012.
44. Waxmonsky J, Kilbourne AM, Goodrich DE, Nord KM, Lai Z, Laird C, Clogston J, Kim HM, Miller CJ, Bauer MS. Enhanced fidelity to treatment for bipolar disorder: results from a randomized controlled implementation trial. Psychiatric Services 2014; 65:81-90.
45. Stetler CB, Legro MW, Rycroft-Malone J, Bowman C, Curran MG, Hagedorn H, Pineros S, Wallace CM. Role of "external facilitation" in implementation of research findings: a qualitative evaluation of facilitation experiences in the Veterans Health Administration. Implement Sci 1:23, 2006.
46. Kirchner JE, Parker LE, Bonner LM, Fickel JJ, Yano EM, Ritchie MJ. Roles of managers, frontline staff and local champions in implementing quality improvement. J Eval Clin Pract 2012; 18:63-69.
47. Kirchner JE, Kearney LK, Ritchie MJ, Dollar KM, Swenson AB, Schohn M. Lessons learned through a national partnership between clinical leaders and researchers. Psychiatric Services 2014; doi: 10.1176/appi.ps.201400054).
48. Kirchner JE, Ritchie MJ, Dollar KM, Gundlach P, Smith JL. *Implementation Facilitation Training Manual: Using External and Internal Facilitation to Improve Care in the Veterans Health Administration.* North Little Rock, Ark, US Department of Veterans Affairs, Health Services Research and Development, Mental Health Quality Enhancement Research Initiative, 2013. On-line. Accessed 8/21/14.
49. Goodrich DE, Bowersox NW, Abraham KM, Burk JP, Visnic S, Lai Z, Kilbourne AM. Leading from the middle: replication of a re-engagement program with veterans with mental disorders lost to follow-up care. Depression Research and Treatment 2012; doi:10.1155/2012/325249.

50. Parchman ML, Noel PH, Culler SD, Lanham HJ, Leykum LK, Romero RL, Palmer RF. A randomized trial of practice facilitation to improve the delivery of chronic illness care in primary care. Impl Sci 2013; 8:93.
51. Jordan ME, Lanha HJ, Crabtree BF, Nutting PA, Miller WL, Stange KC, McDaniel RR. The role of conversation in health care interventions: enabling sensemaking and learning. Implement Sci 2009; 4:15.
52. Plesk, P. Redesigning health care with insights from the science of complex adaptive systems. In: Institute of Medicine, *Crossing the Quality Chasm.* Washington, DC, National Academy Press, 2001, pp 309-322.
53. Bajor LA, Miller CJ, Holmes S, Van Deusen Lukas C, Bauer MS. Collaborative care for returning veterans. J Depr Anxiety 2013; 2:2, doi.org/10.4172/2167-1044.1000134.
54. Miles MB, Huberman AM. *Qualitative Data Analysis: An Expanded Sourcebook*. Thousand Oaks, CA: Sage, 1994.
55. Utarini A., Winkvist A, Pelto GH. Appraising studies in health using rapid assessment procedures (RAP): Eleven Critical Criteria. Human Organization 2001 60(4): 390-400.
56. Hsieh HF, Shannon SE. 3 approaches to qualitative content analysis. Qual Health Res 2005; 15:1277-88.
57. Powell BJ, McMillen JC, Proctor EK, Carpenter CR, Griffey RT, Bunger AC, Glass JE, York JL. A compilation of strategies for implementing clinical innovations in health and mental health. Med Care Res Rev 2011; 69:123-57.
58. Hussey MA, Hughes JP. Design and analysis of stepped wedge cluster randomized trials. Contemp Clin Trials, 2007; 28:182-191.
59. Brown, C. A., & Lilford, R. J. (2006). The stepped wedge trial design: a systematic review. BMC Med Res Methodol, 6, 54. doi: 10.1186/1471-2288-6-54.
60. King G, Gakidou E, Ravishankar N, Moore RT, Lakin J, Vargas M, Tellez-Rojo M, Hernandez Avila JE, Hernandez Avila M, Hernandez Llamas H. A “politically robust” experimental design for public policy evaluation, with application to the Mexican Universal Health Insurance Program. J Policy Analysis and Management 2007; 26:479-506.
61. Gugiu PC, Coryn C, Clark R, Kuehn A. Development and evaluation of the short version of the Patient Assessment of Chronic Illness Care instrument. Chronic Illn 2009 Dec;5(4):268-76.
62. Stevanovic D. Quality of Life Enjoyment and Satisfaction Questionnaire—short form for quality of life assessments in clinical practice: a psychometric study. J Psychiiatr and Ment Health Nurs 2011; 18:744-50.
63. Nabati L, Shea N, McBride L, Gavin C, Bauer MS. Adaptation of a simple patient satisfaction survey to mental health: psychometric properties. Psychiatry Res 1998; 77:51-56.
64. Selim AJ, Rogers W, Fleishman JA, Qian SX, Fincke BG, Rothendler JA, Kazis LE. Updated U.S. population standard for the Veterans RAND 12-item Health Survey (VR-12). Qual Life Res. 2009;18:43-52.
65. Dube P, et al. The P4 Screener: evaluation of a brief measure for assessing potential suicide risk in 2 randomzied effectiveness trials of primary care and oncology patients. *Primary Care Companion to the Journal of Clinical Psychiatry* 2010; 12(6): PCC.10m00978.
