# Supplementary material for: Effectiveness of Implementing a Collaborative Chronic Care Model for Clinician Teams on Patient Outcomes and Health Status in Mental Health: A Randomized Clinical Trial
Source: JAMA Netw Open. 2019 Mar 1;2(3):e190230. doi: 10.1001/jamanetworkopen.2019.0230 (PMC6484628; doi:10.1001/jamanetworkopen.2019.0230)
Supplement: Supplement 2. — eTable. By-Facility Summary of Implementation Outcomes [file jamanetwopen-2-e190230-s002.pdf]

Supplementary Online Content

Bauer MS, Miller CJ, Kim B, et al. Effectiveness of implementing a collaborative chronic care model for clinician teams on patient outcomes and health status in mental health: a randomized clinical trial. *JAMA Netw Open.* 2019;2(3): e190230. doi:10.1001/jamanetworkopen.2019.0230

eTable. By-Facility Summary of Implementation Outcomes

This supplementary material has been provided by the authors to give readers additional information about their work.

©2019 Bauer MS et al. *JAMA Network Open.*

eTable. By-Facility Summary of Implementation Outcomes

	Process Redesign Results % CCM-based processes re/designed	***Team Development Measure: Average % positive responses for Roles Clarity & Team Primacy Subscales***	***Relative Level of Implementation (>70% on both measures)***
**Wave 1 Site A**	89%	93%	Higher
**Wave 1 Site B**	44%	39%	Lower
**Wave 1 Site C**	69%	61%	Lower
**Wave 2 Site D**	72%	73%	Higher
**Wave 2 Site E**	44%	61%	Lower
**Wave 2 Site F**	65%	64%	Lower
**Wave 3 Site G**	85%	98%	Higher
**Wave 3 Site H**	80%	85%	Higher
**Wave 3 Site I**	69%	58%	Lower

Wave denotes order in which facilities received implementation support in the stepped wedge desig. Data column 1 summarizes the proportion of clinical processes reviewed that were concordant with the Collaborative Care Model (CCM) for each facility. Data column 2 summarizes the average percent positive responses for each facility on the Team Development Measure subscales of Role Clarity and Team Primacy. Data column 3 dichotomizes the sample into higher vs. lower implementers defined by scores of >70% on both measures. See text for details on measures and analyses.

©2019 Bauer MS et al. *JAMA Network Open.*
