# Supplementary material for: Effectiveness of Implementing a Collaborative Chronic Care Model for Clinician Teams on Patient Outcomes and Health Status in Mental Health: A Randomized Clinical Trial
Source: JAMA Netw Open. 2019 Mar 1;2(3):e190230. doi: 10.1001/jamanetworkopen.2019.0230 (PMC6484628; doi:10.1001/jamanetworkopen.2019.0230)
Supplement: Supplement 3. — Data Sharing Statement [file jamanetwopen-2-e190230-s003.pdf]

Data Sharing Statement

Bauer. Effectiveness of Implementing a Collaborative Chronic Care Model for Clinician Teams on Patient Outcomes and Health Status in Mental Health. *JAMA Netw Open*. Published March 01, 2019.
10.1001/jamanetworkopen.2019.0230

Data
Data available: No

Additional Information
Explanation for why data not available: VA privacy regulations preclude overall release
